# Gas Chromatography/Mass Spectrometry Chemical Profiling of Volatile Compounds from Cranberry Plant Byproducts as Potential Antibacterials, Antifungals, and Antioxidants

**DOI:** 10.3390/molecules30092047

**Published:** 2025-05-04

**Authors:** Martin Aborah, Frank Scarano, Catherine Neto

**Affiliations:** 1 Department of Chemistry and Biochemistry, University of Massachusetts Dartmouth, North Dartmouth, MA 02747, USA; maborah@umassd.edu; 2 Department of Medical Laboratory Science, University of Massachusetts Dartmouth, North Dartmouth, MA 02747, USA; fscarano@umassd.edu

**Keywords:** *Vaccinium macrocarpon*, GC-MS, berry volatiles, Early Black (EB), Mullica Queen (MQ), Stevens (ST), 2,2-diphenyl-1-picrylhydrazyl (DPPH), antimicrobial, antifungal, hydrodistillation, cinnamaldehyde, terpineol

## Abstract

The increasing resistance of microorganisms to currently used antimicrobials requires the urgent development of new effective treatments. Plant-based natural products can be an alternative solution. The aerial plant parts of the cranberry (*Vaccinium macrocarpon*) present a potential new source of antimicrobial secondary metabolites. Volatile essential oils were extracted from Stevens, Early Black, and Mullica Queen variety plants by steam distillation (SD) and the Clevenger method (CM), and their profiles were characterized by GC-MS. The extracts and two identified constituents, cinnamaldehyde and terpineol, were screened by the disc diffusion assay against Gram-positive *B. cereus* ATCC 11778 and *S. aureus* ATCC 25923 and Gram-negative bacteria *E. coli* ATCC 25922, *P. aeruginosa* ATCC 27853, and *C. albicans* ATCC 14053. Radical scavenging antioxidant activity was also determined using the DPPH assay. The CM extracts were rich in fatty acids, sesquiterpenes, and diterpenes, whereas the SD extracts contained more aldehydes, monoterpenes, and phenylpropanoids. All volatile extracts showed promising antioxidant activity; leaf extract activity was significantly higher than the vine (*p* < 0.05). The CM leaf and vine extracts exhibited antimicrobial activity against *B. cereus*, *S. aureus*, *E. coli*, and *C. albicans* compared to the SD, and the leaf extracts were more effective than the vine extracts. Individual constituents of leaf and vine extracts, cinnamaldehyde and α-terpineol, also showed antimicrobial activity against these organisms. The active constituents of the CM extracts are yet to be identified. A multivariate analysis revealed a particular pattern of inhibition of the tested organisms. Based on our results, cranberry volatile extracts have potential for future valorization as antibacterials, antifungals, and antioxidants.

## 1. Introduction

The increasing resistance of bacteria to antibiotics via their structural modification to prevent the permeability, excretion, and neutralization of the drugs has prompted scientists to search for new biocides to fight bacterial diseases [1]. Bacteria are a major concern to public health due to their emerging resistance to established antibiotics. The World Health Organization categorizes resistant infections as a leading cause of death worldwide among the top ten risks to global health (WHO. WHO World Antimicrobial Awareness Week-WAAW from 18 to 24 November 2020. Available online: https://www.who.int/campaigns/world-antimicrobial-awareness-week/2020 (accessed on 18 May 2024)). The drivers of the resistance are due to the overuse of antimicrobials by humans, and in treating animals and plants. A broad range of both community-acquired and hospital-acquired infections, such as skin and soft tissue infections, osteomyelitis, sepsis, endocarditis, and food poisoning, are caused by the Gram-positive bacterium *Staphylococcus aureus* [2]. Infection control methods such as handwashing and hospital sanitization have been utilized to control the infection but have proved challenging due to resistant strains such as Methicillin-Resistant Staphylococcus Aureus (MRSA). Food, plants, and soil all contain the Gram-positive bacterium *Bacillus cereus*, which produces toxins causing nausea, vomiting, and diarrhea and can also induce septicemia and endophthalmitis, resulting in blindness in immunocompromised hosts (McDowell RH, Sands EM, Friedman H. Bacillus Cereus. [Updated 23 January 2023]. In: StatPearls [Internet]. Treasure Island (FL): StatPearls Publishing; 2024 Jan. Available from: https://www.ncbi.nlm.nih.gov/books/NBK459121/ (accessed on 18 May 2024). A Gram-negative bacillus, *Escherichia coli*, is responsible for diarrheal infections [3]. *E. coli* belongs to the intestinal microbiota of healthy people but can be pathogenic when outside the intestine, especially in urinary tract infections. *P. aeruginosa* infections are extremely difficult due to the microbe’s rapid mutations and adaptation to gain resistance to antibiotics, and they are among the top-listed pathogenic hospital-acquired infections [4]. The development of highly resistant forms of bacteria has outpaced the discovery of new medicines, underscoring the urgent need to create antibiotic substitutes [5]. Keeping in mind the safety and health concerns of synthesized drugs, there is a search for natural products as alternatives.

One such alternative is the volatile compounds in the essential oils of cranberry leaf and vine aerial parts. The traditional medicinal use of the cranberry plant has been documented: the plant is steeped as medicine for pleurisy, boiled and rubbed in sore eyes by indigenous people [6], and ingested as tea for nausea [7]. The health benefits of berries are well documented due to their rich content of phytochemicals, including polyphenols and terpenes, which are essential for human health. Cranberries have received much attention as they are a rich source of phenolic compounds, primarily flavonols, proanthocyanidins, and anthocyanins [8]. Several in vitro and in vivo studies have demonstrated properties of cranberry extracts, including antioxidant (scavenging free radicals) [9,10,11,12], anti-inflammatory (suppressing COX enzymes, cytokines, and NO production) [8,13,14], antiviral [15,16], antibacterial [17,18], and anticancer properties [19,20], largely attributed to the availability of the phenolic compounds, which are well established in the literature for their biological properties.

Volatile phytochemicals are also present in the cranberry plant but have received little attention as potential bioactive constituents. They are small and low molecular weight compounds (below 250–300 Da) with high vapor pressure due to their low boiling point [21,22]. The volatiles content of berries varies depending on a number of factors, such as temperature, experimental circumstances, fruit samples, maturity, and pre- and post-harvest storage conditions [23]. Volatiles consist of diverse classes of compounds, including terpenes and terpenoids (mostly monoterpenes, sesquiterpenes, and diterpenes), esters, alcohols, acids, aldehydes, ketones, fatty acids, phenylpropanoids, and some aromatic compounds. Berry volatiles are developed during the maturation and ripening stage of development [24] and function as allelochemicals and semiochemicals. Unlike berry polyphenols, information on the health benefits of the volatile compounds as antioxidants, and particularly as antimicrobials, is limited, as there are no published data to the best of our knowledge. Few articles published in the literature focus on the chemical composition, sensory quality, and evaluation of experimental parameters such as effective extraction methods [25]. Many researchers have agreed on the fact that monoterpenes are abundant among the volatile compounds in cranberry fruit, but chemical profiling of the leaf and vine volatiles has not yet been reported. The monoterpenes are synthesized through the mevalonic acid (MVA) pathway in the cytosol and the 2-methylerythritol 4-phosphate (MEP) pathway (by generating isopentenyl diphosphate (IPP) and dimethylallyl pyrophosphate (DMAPP) precursors) [26]. There are reports on the anti-inflammatory [21,27], antioxidant [27,28], anticancer [29], and protein-binding abilities [30] of berry volatiles, but information on the antimicrobial ability of the volatiles is scarce in the literature [28]. Moreover, limited research exists on volatiles extracted from different cranberry cultivars, other parts of the cranberry plant, or a combination of different *Vaccinium* species [31].

To fill the current research gap in the area, we aimed to study the chemical composition of volatile extracts from the aerial parts of three cranberry cultivars: Early Black (EB), Stevens (ST), and Mullica Queen (MQ). We aimed to establish a thorough chemical profiling of extracts prepared by either steam distillation (SD) or modified Clevenger (CM) extraction. We hypothesized that the cranberry plant volatile extracts could exhibit antioxidant and antimicrobial properties against two Gram-positive bacteria (*S*. *aureus* and *B*. *cereus*), two Gram-negative bacteria (*E. coli* and *P. aeruginosa*), and a representative yeast (*C. albicans*) strain, which can be pathogenic under uncontrolled conditions. The current research is the first report of the chemical characterization of volatile compounds of cranberry aerial parts and their antimicrobial activity. The research is useful to the food industry and agricultural crop cultivation.

## 2. Results and Discussion

### 2.1. Chemical Profiling

The tentative identification of these compounds was confirmed using the NIST/EPA/NIH Mass Spectral Library version 2.0 (search and reverse search index of 800), the literature, a comparison with published literature by ADAMS [32], authentic standards, and the linear retention index using a series of n-alkanes (C7-C40) (Tables S1 and S2 and Figure S1). Representative chromatograms of the cranberry leaf and vine cultivars are presented in Figures S2–S5. The identified compounds were mainly distributed in ten family classes, namely monoterpenes, sesquiterpenes, diterpenes, phenylpropanoids, fatty acids, ketones, aldehydes, aromatic hydrocarbons, esters, and alcohols. The cranberry leaf and vine volatile profile under different extraction methods showed that there was a significant difference between the number and distribution of compounds (*p* < 0.05) (Table 1 and Table 2). Similarly, Karimnejad et al. (2024) reported differences in the essential oil compounds of *Mentha longifolia* under the Clevenger vs. steam distillation methods [33]. Studies of other plants have shown that the extraction technique has a significant effect on the profile of plant essential oils [34,35]. Within a single extraction technique, we also observed some diversity among the different cultivars. Specifically, there were some differences in the compounds produced using the Clevenger or steam distillation technique for both the leaves and vines of the various cultivars. Many factors may account for variation in the secondary metabolites, including seasonal variation and the specific genotype of the cultivar. Although they possess many common compounds, each cranberry leaf and vine cultivar has its own distinct chemical fingerprint. The Clevenger method extracted more fatty acids than steam distillation, but both methods extracted sesquiterpenes and diterpenes. The steam distillation method extracted more monoterpenes and phenylpropanoids [35].

Trans-cinnamaldehyde was identified as a major component of both EB leaf (12.8%) and MQ leaf (27.8%) steam distillation extracts, followed by benzenepropanal (14.2%, 17.8%), α-terpineol (4.97%, 16.4%), 2-Decenal (6.13%, 5.25%), and phytol (1.79%, 4.00%) for EB and MQ leaf, respectively. Due to a lack of sufficient plant material, we were unable to prepare and analyze extracts of the Stevens variety leaf or vine by steam distillation. Thus, it is unclear whether the compounds noted above are also major components of Stevens foliage, with the exception of phytol, which was detected in CM extracts of all three cultivars as discussed below. In contrast to the cranberry leaf, Eudesm-7(11)-en-4-ol (49.8%, 22.9%), α-terpineol (8.39%, 25.2%), myrtenol (2.27%, 9.28%), γ-selinene (1.89%, 3.26%), and nonanal (1.78%, 2.81%) were the principal components in EB and MQ vine steam distillation extracts, respectively. In this case, a comparison with the literature can only be made for the essential oils of other plants, as this is the first attempt to characterize the volatile essential oil profile of these aerial parts of the cranberry plant. Cinnamaldehyde has been reported as 33.9% in cinnamon leaf essential oil [36], possesses no toxicity to animals and humans, and is known as a biologically active component [37]. Concerning some variation in secondary metabolites, it is interesting to note that EB leaf contains benzaldehyde (1.87%), as well as benzeneethanol, β-ethenyl-α-methyl- (1.59%), which was rather absent in MQ leaf. In contrast, p-Ethylguaicol (6.64%) was predominant only in the MQ vine.

Using the Clevenger method, both the leaf and vine extracts primarily comprised fatty acids, sesquiterpenes, diterpenes, aldehydes, and alcohols. Docosane (1.30–20.9%), phytol isomer (1.14–13.8%), and 7-oxanorbornene (trace-51.5%) varied substantially between the three cultivars. The variation in 7-oxanorbornene among cultivars could be due to a response to pest infestation [38,39], which can be localized to specific parts of a bog. Moreover, it is reported that certain cultivars are more resistant to pest infestation than others [40]. As volatiles often play a signaling role in plants or are produced in response to environmental stressors such as pests or fungal infestation, secondary metabolites can vary widely between plant samples. Secondary metabolite composition in cranberry foliage can be cultivar-dependent; for example, the variation in coumaroylquinic acids and quercetin derivatives between Early Black and Howes cultivars that was reported previously [41]. Other compounds identified in CM leaf extracts were phytol (2.89–8.76%), kaur-16-ene (5.06–6.27%), methyl palmitate (1.76–4.20%), octadecanol (5.06–5.58%), tetradecanoic acid (trace-10.8%), hexadecenoic acid (4.44–4.54%), 2-methyl-7-octadecyne (0.55–4.99), tridecene (2.70–3.36%), heptadecane (trace-2.91%), and hexadecene (0.73–2.93%). The dominant compounds in the CM vine extracts were methyl linoleate (2.34–16.6%), docosene (3.05–19.3%), and Labd-(13E)-8,15-diol (23.9–63.6%), which varied substantially between cultivars. Other constituents include phytol (0.40–2.04%), heneicosane (1.63–2.28%), eicosene (2.72–6.25%), hexadecenoic acid (trace-12.8%), eudesm-7(11)-en-4-ol (1.20–3.33%), and tridecanol (0.88–3.34%). The choice of extraction technique also influenced the biological properties of the extracts. As such, we observed greater antimicrobial activity of the essential oils extracted by the Clevenger method compared to the steam distillation method. Together, the two extraction techniques produce a novel fingerprint of volatiles present in cranberry leaf and vine, with each cultivar presenting a distinct fingerprint that could be essential to their biological behavior.

### 2.2. Antioxidant Activity

**Figure 1:**
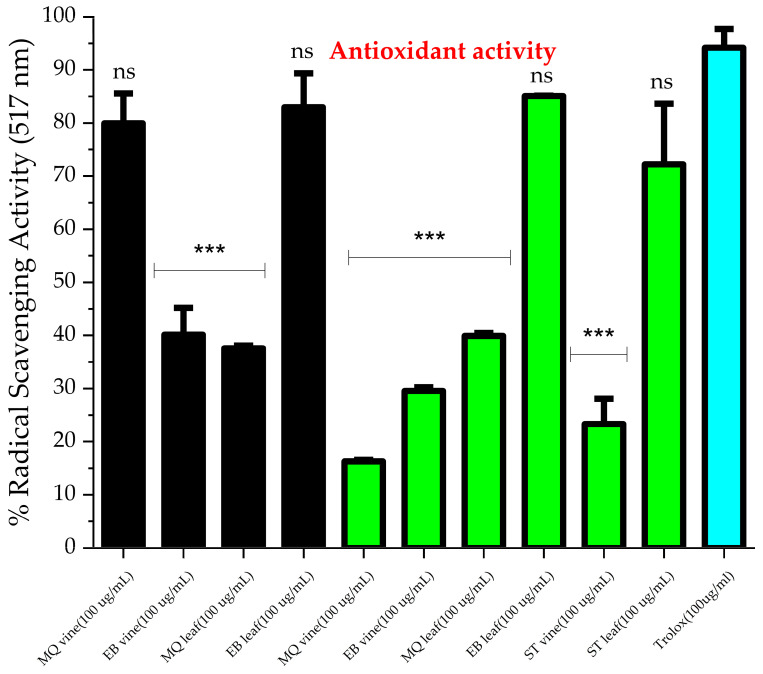
DPPH antioxidant activity of cranberry leaf and vine extracts produced by steam distillation method (black color) and Clevenger method (green color), compared to standard antioxidant Trolox, all at 100 µg/mL. Data are presented as mean ± standard deviation of triplicates. DPPH activity was not conducted for ST vine and leaf under steam distillation technique due to inadequate sample. *** *p* < 0.001 and significantly lower than positive control. ns = not significantly different from positive control.

**Figure 2:**
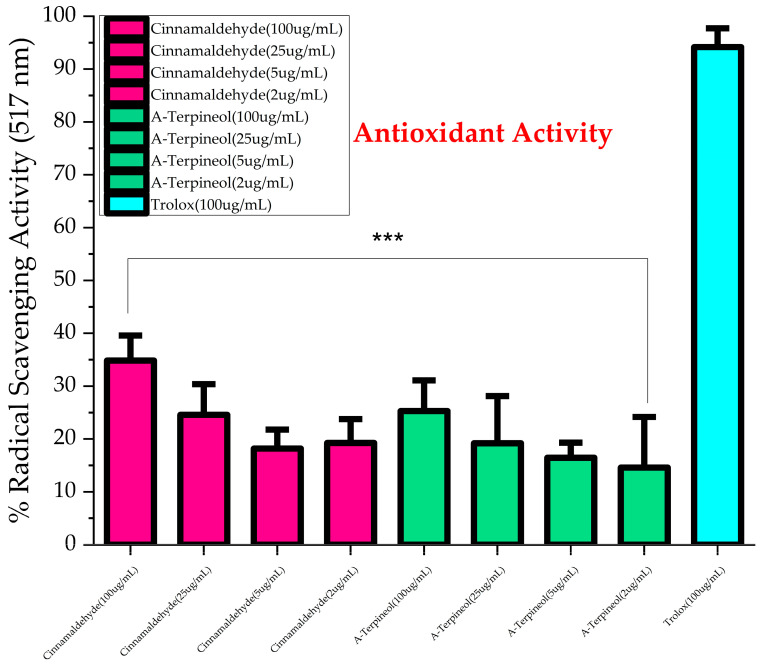
DPPH scavenging antioxidant activity of alpha terpineol and cinnamaldehyde, major components of steam distillation extracts at 100, 25, 5, and 2 µg/mL, compared to standard antioxidant Trolox at 100 µg/mL. Data are presented as mean ± standard deviation of triplicates. *** *p* < 0.001 and significantly lower than positive control.

The free radical scavenging ability of the cranberry aerial parts was assessed by the DPPH scavenging assay (Figure 1) for cranberry leaf and vine essential oils extracted using both methods. Comparing the two extraction techniques, the pattern of the antioxidant activity of the cranberry leaf extracts was similar; nevertheless, the EB leaf cultivar was significantly higher than the MQ cultivar (*p* > 0.05). Some variation in the antioxidant activity of the cranberry vine extracts was observed between techniques. We believe this is the first report on the antioxidant activity of cranberry leaf and vine volatile extracts. Considering specific cultivars, the MQ vine antioxidant activity was greater for the steam distillation extract compared to the Clevenger method extract (*p* < 0.05), whereas similar radical scavenging behavior was observed for the two EB vine extracts. EB leaf (SD and CM extracts), ST leaf (CM extract), and MQ vine (SD extract) were statistically similar in efficacy to the positive control Trolox at 100 µg/mL. The remaining extracts were significantly less effective than Trolox at 100 µg/mL. The variation in composition of the extracts between cultivars and extraction techniques could account for these results. p-ethylguaicol and m-eugenol, which were detected in steam-distilled MQ vine extract, could account for the antioxidant behavior. In a previous study, these compounds were reported to relieve oxidative stress induced in a HepG2 cell model [42] and block lipid peroxidation by the hydroxyl radical [43], respectively. Moreover, the antioxidant activity of the steam-distilled EB leaf could be explained by the presence of megastigmatrienone. A study reported the potent antioxidant activity of *Tecoma smithii* Will. Wats leaves, which were abundant in megastigmatrienone [44]. The observed behavior indicates a capacity of the volatile extracts to scavenge free radicals. The potential of these essential oils for free radical inhibition may involve specific compounds eliminating free radical formation, such as those discussed, or possibly synergistic activity between several components [45]. Due to the predominance of cinnamaldehyde and A-terpineol produced by the steam distillation method and prior bioactivity reported in the literature, these two compounds were screened for DPPH scavenging and antimicrobial activity. Both compounds exert a mild antioxidant effect compared to that of Trolox at 100 µg/mL. We found no significant difference between the antioxidant behavior of α-terpineol and trans-cinnamaldehyde (*p* = 0.444) (Figure 2) at the concentrations tested. A-terpineol has been reported to exhibit antioxidant behavior by the DPPH assay [46] but less than that of conjugated monoterpenes. The conjugation increases the antioxidant behavior of the monoterpenes. To function as a direct antioxidant, a novel radical generated during the reaction with DPPH• must not perpetuate the chain reaction. The antioxidant radical is stabilized through electron delocalization by the π bonds, thus terminating the chain reaction [47]. A-terpineol is not conjugated, thus explaining the observed behavior (26.3 ± 2.8%) at 100 ug/mL in our work. Nonetheless, α-terpineol may contribute significantly to the DPPH scavenging ability of cranberry volatile extract [21,47]. Trans-cinnamaldehyde has been reported to reduce DPPH radical scavenging activity by less than 10% at 100 µg/mL [48]. We found a percent inhibition of 34.8 ± 2.6% at this concentration. A-terpineol showed a lower DPPH scavenging capacity than trans-cinnamaldehyde, with IC_25_ values of 82.5 µg/mL and 37.7 µg/mL, respectively.

We were unable to screen the major constituents of the Clevenger extracts; however, the observed free radical scavenging activity could be due to the presence of compounds such as hexadecanoic acid [49] and phytol [50] or synergistic behavior among constituents. Hexadecanoic acid isolated from *Ipomoea eriocarpato* leaves was capable of scavenging DPPH free radical in a dose-dependent manner [51]. Among the Clevenger extracts, EB leaf and ST leaf were both richer in phytol than the less effective antioxidant MQ leaf extract; phytol has been previously reported as an effective hydroxyl radical scavenger [52].

### 2.3. Antimicrobial Screening

**Figure 3:**
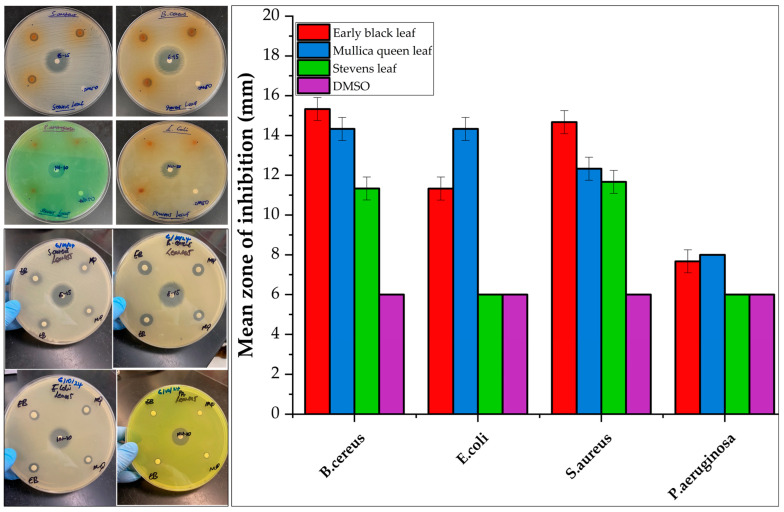
Inhibition of *S. aureus*, *B*. *cereus*, *E*. *coli*, and *P. aeruginosa* by Clevenger extracts at a dose of 10 mg over 24 h, as measured by disc diffusion assay. Data are presented as mean ± standard deviation of triplicates. NB: Mean zone of inhibition of 6 mm means no inhibition.

**Figure 4:**
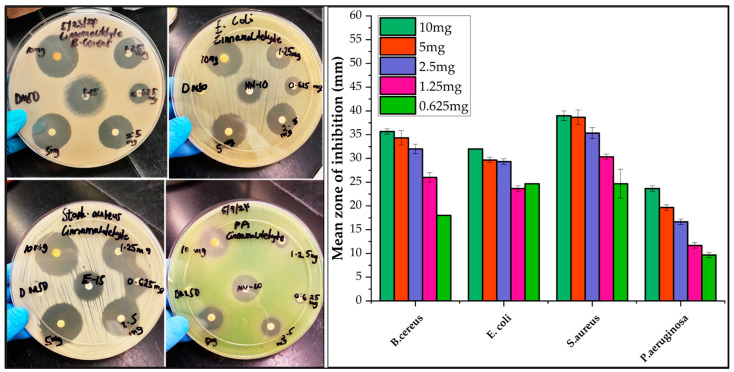
Mean zone of inhibition of test organisms by trans-cinnamaldehyde, a major component of the leaf steam distillation extract. Data represent triplicate analysis and are represented as mean ± standard deviation.

**Figure 5:**
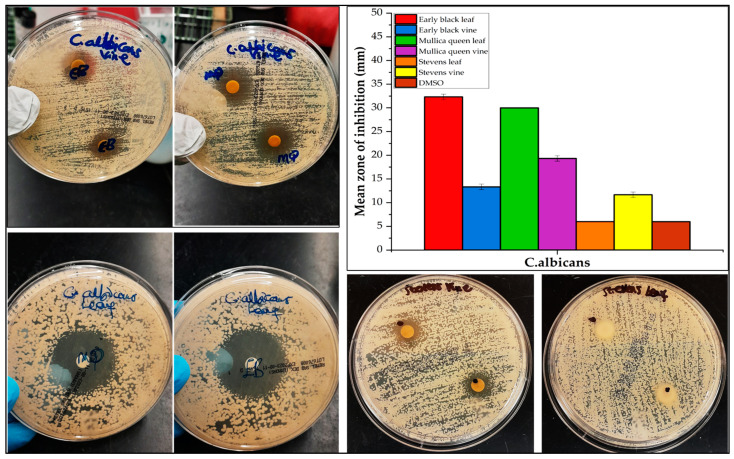
Mean zone of inhibition of *C*. *albicans* ATCC 14053 by cranberry volatile extracts (Clevenger extraction method) at a dosage of 10 mg. Data represent triplicate analysis and are represented as mean ± standard deviation.

**Figure 6:**
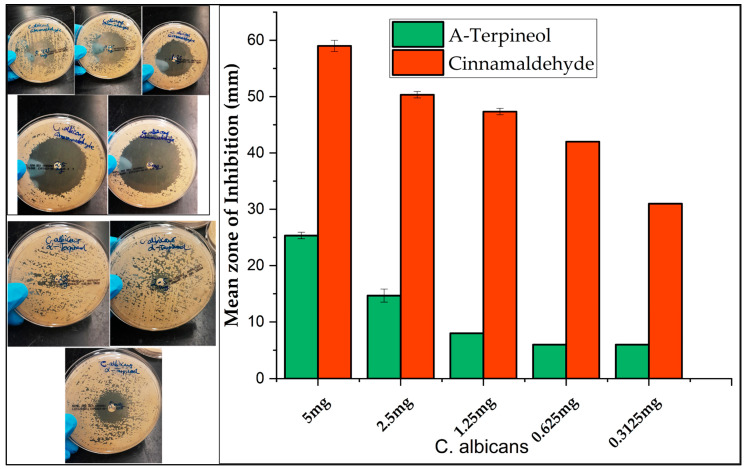
Mean zone of inhibition of *C*. *albicans* ATCC 14053 by trans-cinnamaldehyde and alpha terpineol, compounds detected under the steam distillation method. Data represent triplicate analysis and are represented as mean ± standard deviation.

The antibacterial and antifungal activities of the cranberry leaf and vine volatile essential oils and the individual major constituents from the steam distillation method in each plant part were assessed by the disc diffusion assay against two Gram-negative (*E. coli, P. aeruginosa*) and two Gram-positive (*S. aureus*, *B. cereus*) bacteria, as well as one fungus (*C*. *albicans*) (Figure 3, Figure 4, Figure 5 and Figure 6, Figures S6 and S7). The comparison of the antimicrobial activity from the different extraction techniques is presented in Table 3 along with data from positive controls. The findings revealed that some of the cranberry volatile essential oils caused selective inhibition among the bacteria and fungi tested. The positive controls, erythromycin (0.015 mg) for Gram-positive strains and tobramycin (0.01 mg) for Gram-negative strains, were within acceptable quality control limits. There was no inhibition from pure DMSO as the negative control. The antimicrobial activity of the Clevenger extracts was better compared to the steam distillation extracts. The Clevenger extraction technique produced an oil that is abundant in diterpenes and fatty acids, which could contribute to the antimicrobial behavior. For the Clevenger extracts, measurable bacterial inhibition was observed for most, although it was less than that of positive controls. Inhibition zone diameters for the different leaf cultivars against the bacteria were similar (*p* > 0.05), except for *E. coli and P. aeruginosa,* where ST demonstrated a lower activity compared to the other cultivars. The leaf and vine extracts were active against *C. albicans*, exhibiting greater inhibition than the positive control miconazole nitrate (0.01 mg), with the exception of ST leaf, which did not appear to inhibit *C. albicans*. All leaf and vine extracts showed a weaker antimicrobial behavior against *P. aeruginosa* than the other organisms. Moreover, the inhibition of *E. coli* varied between EB, MQ, and ST leaf. In addition, selective bacterial inhibition was also seen for *S. aureus* and *B. cereus*; the leaf was more antimicrobial than the vine extracts. The variation in the composition of the leaf cultivars could affect antimicrobial activity. Particularly, 7-oxanorbornene [53], heptadecanoic acid [54,55], and linoleic acid [55], which were detected in the EB and MQ but not in ST leaf, could contribute to the observed antimicrobial behavior based on prior reports in the literature. In contrast, the antimicrobial behavior could also be due to compounds not detected and reported in the composition. For the vine cultivars, the observed antimicrobial behavior could be due to the diterpene Labd-(13E)-8,15-diol, which was present in all cultivars in significant quantity [56]. In general, the leaf and vine extracts demonstrated antibacterial and antifungal behavior, suggesting a potential valorization of these byproducts. The antimicrobial activities from the cranberry aerial parts, particularly the leaf extracts, is promising, suggesting that further investigation of their activity on other pathogenic microbes would be useful. However, given the diversity of secondary metabolites identified, a limitation of this study is that we cannot confirm which of these metabolites is responsible for the observed activities. Future studies should focus on confirming the identities of the bioactive constituents of each Clevenger extract that contributed to the observed antimicrobial activities using the bioautography method [57].

When tested individually, the leaf component trans-cinnamaldehyde and terpineol [24], one of the major constituents in the fruit, demonstrated promising activity against *B. cereus, S. aureus,* and *C. albicans* (Figure 4). Cinnamaldehyde has been reported to be effective against pathogenic *E. coli* strain 042 and is a leading compound in the development of antibacterial agents [58]. Our work recorded a higher mean zone of inhibition for cinnamaldehyde than α-terpineol (*p* < 0.05). Shu et al. (2024) reported a similar pattern of inhibition for cinnamaldehyde [59], with *C. albicans* being the most sensitive. For the current work, the IC_50_ value of cinnamaldehyde for *C. albicans* was 2.77 µg/mL, suggesting its potency against the organism. Cinnamaldehyde presented better antimicrobial activity, with IC_30_ values of 0.771 µg/mL (*S. aureus*), 3.57 µg/mL (*B. cereus*), and 5.12 µg/mL (*E. coli*). Cinnamaldehyde produced a steeper log phase on the growth curve of *P. aeruginosa* compared to the other bacteria, thus explaining the resistant behavior of the organism (Figures S8 and S10). *P*. *aeruginosa* demonstrated resistance compared to the other organisms. Wang et al. (2019) has reported the MICs and MBCs of α-terpineol for *E. coli* (0.747, 0.747 mg/mL) and *S*. *aureus* (1.868, 1.868 mg/mL) [60], which contrasts with our results (Table 4); however, the strain was not reported in their study. We observed a moderate mean zone of inhibition for α-terpineol in all tested organisms, with a non-concentration-dependent optical density (OD_600_ nm) growth curve (Figure S10). Therefore, the IC values for α-terpineol were not calculated. Interestingly, α-terpineol demonstrated excellent growth inhibition (0.78 µg/mL) for *E*. *coli* CMCC (B) 44102 strain. Electron microscopy revealed decreased cell size, irregular cell shape, and ruptured cell wall and cell membrane in another work [61], suggesting that the strain of bacteria utilized in their experiment was sensitive. Different strains of organisms may possess different resistance abilities.

### 2.4. Multivariate Analysis

**Figure 7:**
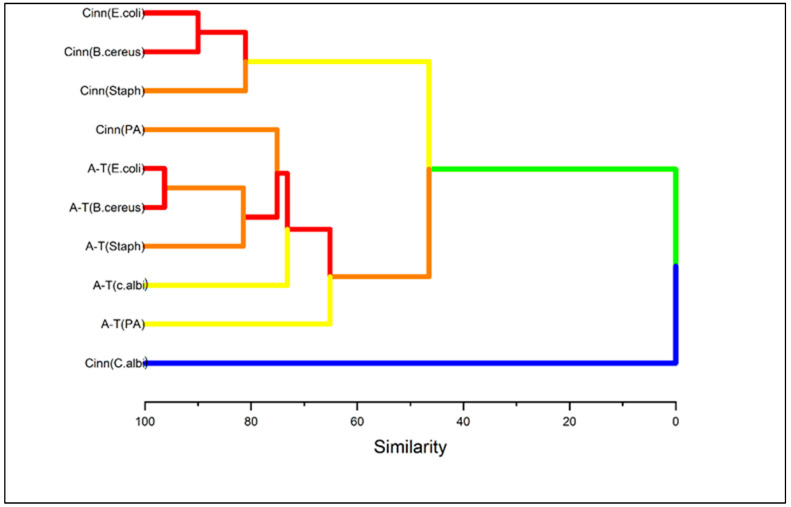
Hierarchical cluster analysis dendrogram of trans-cinnamaldehyde (Cinn) and α-terpineol (A-T) against inhibition of tested organisms. The variables were the mean zone of inhibition. Cluster analysis was conducted to reveal the pattern of inhibition of the bacteria and fungi by cinnamaldehyde and α-terpineol. The cluster method was based on the furthest neighbor, and the distance was calculated by Euclidean type. The Y-axis was reported as similarity among the clusters.

**Figure 8:**
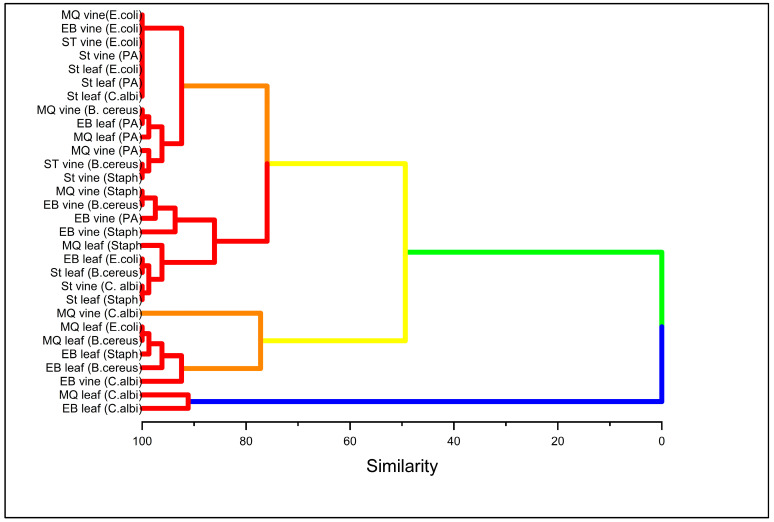
Hierarchical cluster analysis dendrogram of cranberry leaf and vine cultivars against inhibition of tested organisms. The variables were the mean zone of inhibition. Cluster analysis was conducted to reveal the pattern of inhibition of the bacteria and fungi by the cranberry samples. The cluster method was based on the furthest neighbor, and the distance was calculated by Euclidean type. The Y-axis was reported as similarity among the clusters.

The dendrogram from the hierarchical cluster analysis offers a valuable insight for identifying patterns of similarities among the inhibition of the organisms by cinnamaldehyde and α-terpineol (Figure 7) and cranberry leaf and vine samples (Figure 8). In Figure 7, the hierarchical cluster analysis based on the Euclidean distance between groups showed three major clusters [63]. The first cluster, characterized by the similarity of two sub-groups, can be subdivided into Cinn (*E. coli*), Cinn (*B. cereus*), and Cinn (*S. aureus*). The second cluster consists of five similar sub-groups, namely Cinn (*P. aeruginosa*), A-T (*E. coli*), A-T (*B. cereus*), A-T (*S. aureus*), A-T (*C. albicans*), and A-T (*P. aeruginosa*). Cinn (*C. albicans*) comprises the third cluster, which is distinct from all the others. Notably, the cluster analysis revealed a similar pattern of cinnamaldehyde inhibition against *E*. *coli* and *B*. *cereus*, and α-terpineol against the same organisms. It is noteworthy that the cinnamaldehyde inhibition of *P*. *aeruginosa* was classified under group 2 among α-terpineol inhibitions, suggesting the resistant behavior of the organism. Of particular interest is the cinnamaldehyde inhibition against *C*. *albicans,* which was dissimilar to all the other groups, demonstrating its efficacy against the fungal strain. The cluster analysis for the cranberry samples revealed four major clusters with sub-groups (Figure 8). The first cluster consists of two sub-groups; the first sub-group, consisting of seven individual members, showed the lowest inhibition pattern against the test organisms. Particularly, *E. coli* demonstrated resistance to all the cranberry vine cultivars, in addition to ST leaf. The second cluster consists of two sub-groups that showed a common inhibition pattern of *S. aureus* among MQ and ST leaf, which was different from the EB leaf, which is classified under the third cluster. We observed MQ and EB leaf clustered under a common sub-group within a cluster, showing some similarity in their antimicrobial ability against the tested organisms. Specifically, sub-group 2 of cluster 1 shows some similarity in their inhibition against *P. aeruginosa*, sub-group 2 of cluster 3 shows some common inhibition against *B. cereus*, and cluster 4 against *C. albicans*. Sub-group 2 of cluster 3 shows the exception, where MQ leaf demonstrated efficacy against *E. coli* and EB against *S. aureus*, despite their common sub-group. Through multivariate analysis, cluster analysis revealed three major inhibition patterns for cinnamaldehyde and α-terpineol and four inhibition patterns for the cranberry leaf and vine extracts.

### 2.5. Microscopy of Treated Bacteria

**Figure 9:**
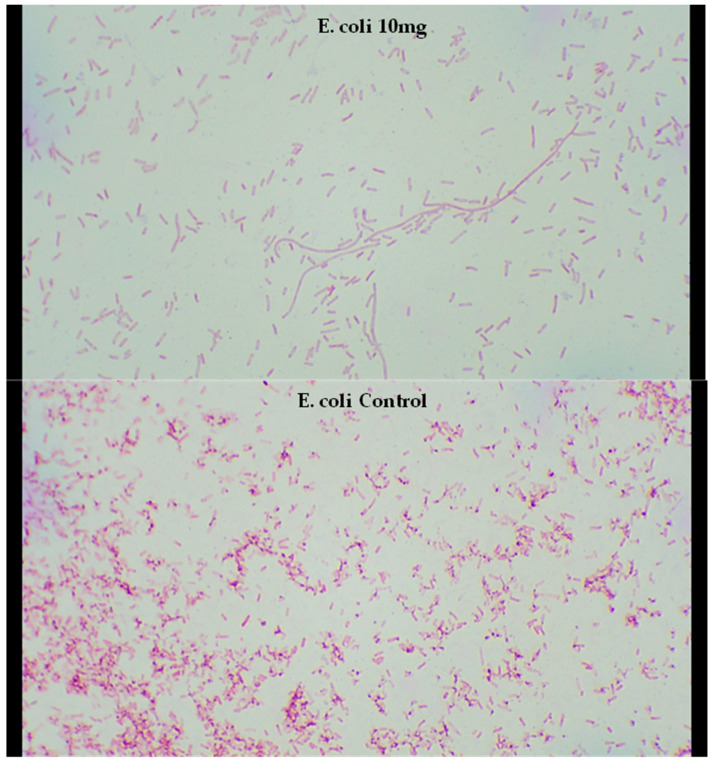
Light microscope examination of the morphological changes of *E*. *coli* treated by trans-cinnamaldehyde (**top**) at 10 mg/mL and *E. coli* control (**bottom**). The microscope was operated at the highest magnification (1000×).

**Figure 10:**
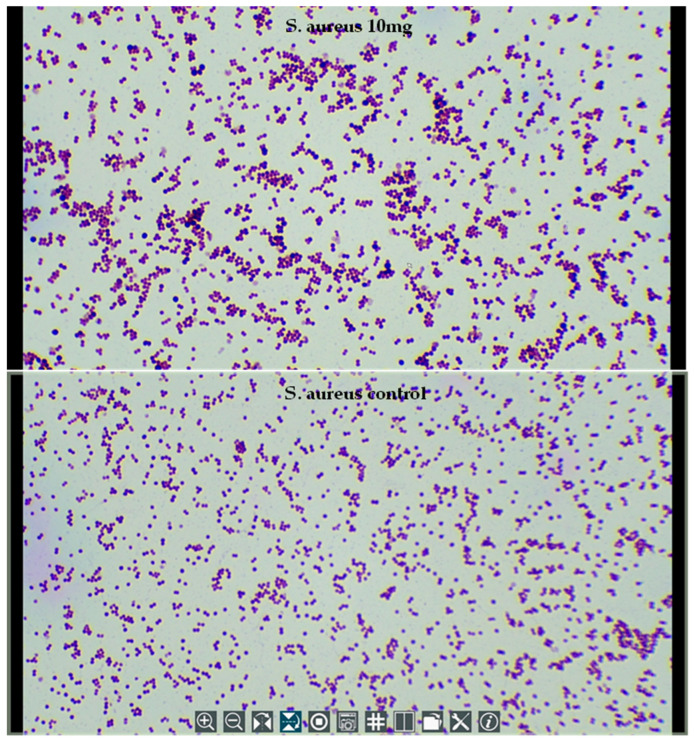
Light microscope examination of the morphological changes of *S*. *aureus* treated by trans-cinnamaldehyde at 10 mg/mL (**top**) and *S. aureus* control (**bottom**). The microscope was operated at the highest magnification (1000×).

Due to the excellent efficacy of trans-cinnamaldehyde against the bacteria and fungi strains tested, the highest inhibiting concentration (10 mg/mL) was observed by light microscope to investigate the morphological changes in the appearance of the cells of one Gram-negative and one Gram-positive type of bacteria after the treatment (Figure 9 and Figure 10). The results revealed a possible elongation of the *E*. *coli* cells compared to the bacterial control. Compared to the published literature, sublethal treatment at 200 mg/L cinnamaldehyde inhibited the growth of *E*. *coli* O157:H7 at 37 °C and for <2 h caused cell elongation [64]. Moreover, cinnamaldehyde’s carbonyl group is predicted to bind to proteins and prevent the bacterial amino acid decarboxylases from exhibiting their activity [36]. *E*. *coli* O157:H7 was able to convert cinnamaldehyde into the less toxic cinnamic alcohol using dehydrogenase/reductase enzymes [64], thus suggesting the antimicrobial activity of cinnamaldehyde could be attributed to the presence of the carbonyl group. The effect of cinnamaldehyde on *S*. *aureus* cells is not clearly visible under a light microscope. Nevertheless, a detailed analysis revealed a non-uniformity of the bacterial cells, where certain cells appear larger compared to the others.

## 3. Materials and Methods

### 3.1. Plant Materials and Chemicals

Cranberry leaf and vine byproduct cultivars (Early Black, Stevens, and Mullica Queen) were obtained from UMass Cranberry Station in East Wareham, MA, USA, in September of 2023. Each cranberry cultivar was hand-harvested into Ziploc storage containers and transported to the laboratory. Care was taken to avoid mixing of the cultivars, which can affect experimental results, and samples with signs of physical damage were rejected. Leaves were manually removed from the vines and separated for storage. The samples were freeze-dried under liquid nitrogen and kept at 4 °C until extraction. GC-MS hexane solvent was obtained from ThermoFisher Scientific (Waltham, MA, USA), and ethanol and HPLC-grade water from Honeywell (Morristown, NJ, USA). An A-terpineol standard was obtained from Extrasynthese (Genay Cedex, France) and Sigma Aldrich (St. Louis, MO, USA). Trans-cinnamaldehyde, C7-C40 saturated alkane mixture and sodium chloride were purchased from Sigma-Aldrich (St. Louis, MO, USA).

### 3.2. Microbial Strains

All the tests were performed on *E. coli* ATCC 25922, *P. aeruginosa* ATCC 27853, *S. aureus* ATCC 25923, *B. cereus* ATCC 11778, and *C. albicans* ATCC 14053 (KwikStik Microbiologics, Saint Cloud, MN, USA). All tested microorganisms were generously donated by the Medical Laboratory Science department, University of Massachusetts Dartmouth, USA. The lyophilized pellet organisms were stored at 4 °C. The bacterial microorganisms were grown on 5% sheep blood agar plates (Remel, Lenexa, KS, USA), and the yeast were grown on Sabouraud Dextrose Agar (Remel, USA). The bacteria were incubated at 37 °C for 24 h, and for yeast, 30 °C for 2 days. A fresh culture was obtained prior to each analysis.

### 3.3. Isolation of Cranberry Volatiles by Hydrodistillation Technique

An extraction procedure was optimized for the analysis of the volatiles. In brief, cranberry leaves and vines were thawed at room temperature, and those with any physical damage were excluded. Approximately 25 g (wet weight) of the samples was blended with 5 g NaCl for 30 s, and the musts were transferred into a 500 mL simple distillation flask using 100 mL of water. The distillation was performed for approximately 1 h at 100 °C. The collecting flask was immersed in an ice-cold water bath, and the distillation set-up was covered with aluminum foil to ensure the trapping of adequate volatiles in the flask. The water–volatile distillate was partitioned into 15 mL of hexane solvent. The procedure was repeated with an additional 15 mL of hexane solvent, and the water fraction was backwashed with an additional 10 mL of hexane. All hexane fractions were collected in a vial, passed through a small column of drying agent, and concentrated through a gentle stream of nitrogen to 5 mL, which was kept at −20 °C until chemical profiling analysis (steam distillation method). The modified Clevenger extraction procedure was followed with 300 mL EtOH at the boiling point. After solvent removal, the cranberry volatile essential oil was kept at −20 °C. Replicate extraction was performed in each case on independent days.

### 3.4. GC-MS Method Development and Optimization

A GC-MS method was developed and optimized for the analysis of the cranberry volatiles. A Focus ITQ Thermo Scientific GC-MS equipped with an AI 3000 auto injector and Xcalibur software 3.1 was employed for the analysis of the samples. The column was TQ-SQC (non-polar capillary column) with dimensions 30 m × 0.25 mm × 0.25 µm (ThermoFisher Scientific, Waltham, MA, USA). The injection port was set in split mode (hot injection technique) with a split flow and ratio of 10 at 250 °C. The carrier gas was helium with a constant flow rate of 1 mL/min. The electron energy was 70 eV. Transfer line and ion source temperatures were 250 and 200 °C, respectively. Fragmentation data from *m*/*z* 25 to 250 were collected in scan mode. The short *m*/*z* range was chosen to increase detector sensitivity and due to the molecular mass of the desired compounds. The solvent delay was set to 3 min. The initial oven temperature was 50 °C. After holding for 3 min, the oven temperature ramped to 100 °C at a rate of 5 °C/min with a hold time of 1 min, then increased to 250 °C at a rate of 10°C/min with a hold time of 1 min. The equilibration time was set at 3 min. The tentative volatile compounds were identified by the GC-MS database NIST/EPA/NIH Mass Spectral Library version 2.0, the literature, and comparison with the published literature by ADAMS [32] and authentic standards, and they were confirmed by calculating the linear retention index (LRI) of each compound using the temperature programming analysis according to the Van den Dool and Kratz equation [65] related to the homologous series of n-alkanes (C7–C40) under the same conditions.

### 3.5. Antioxidant Activity Determination

Antioxidant activity was determined using the DPPH assay (Sigma-Aldrich, St. Louis, MO, USA) with a 96-well plate (Costar Assay plate, polystyrene non-treated, ThermoFisher Scientific, Waltham, MA, USA) [66]. Trans-cinnamaldehyde and alpha terpineol were prepared in ethanol (2, 5, 25, and 100 ppm), crude cranberry volatile essential oils (100 µg/mL), Trolox (100 µg/mL), and DPPH reagent (100 µg/mL). A 25 µL DPPH reagent was added to 100 µL of sample and incubated for 30 min in the dark, and absorbance was read at 517 nm using a spectrophotometer (SpectraMAX 190) with SoftMax Pro 7.0 software. The negative control was a mixture of 100 µL ethanol and 25 µL DPPH, and ethanol was used as a blank. The test was conducted in triplicate in each case.

### 3.6. Disc Diffusion Assay for Antimicrobial Screening

The antimicrobial screening was carried out by the disc diffusion assay technique using a modification of a published method [28]. Under aseptic conditions, fresh microbial cultures were adjusted to 0.5 McFarland Standard turbidity with Mueller Hinton II Broth (Becton, Dickson and Company, Sparks, MD, USA), 10^8^ CFU/mL for bacteria and 10^6^ CFU/mL for yeast. The culture suspensions were aseptically spread on a 150 mm Mueller Hinton II Agar plate (MHA) (USA) with a swab for bacteria. For yeast, 100 µL of suspension was spread on 100 mm Sabouraud Dextrose Agar (SDA) plates. Then, 10 µL of cranberry extracts (10 mg final dosage) and trans-cinnamaldehyde or alpha terpineol (10, 5, 2.5, 1.25, 0.625 mg final dosage) (leading compounds in the steam distillation method) were pipetted onto 6 mm blank sterile paper discs (Sigma Aldrich, USA), which were placed on the inoculated lawn (final concentration on disc). The positive controls for bacteria were erythromycin (E-15) at 0.015 mg (*S*. *aureus*, *B*. *cereus*), tobramycin (NN-10) at 0.01 mg (*E. coli* and *P*. *aeruginosa*), and miconazole nitrate salt (MN-10) at 0.01 mg (*C. albicans*), and DMSO was the negative control. The bacterial plates were incubated at 37 °C for 24 h, while the fungal plates were incubated at 30 °C for 48 h. The widths of the inhibitory zones were measured in millimeters after incubation, and the findings were expressed as mean and standard deviation of three replicates.

### 3.7. Minimum Inhibitory and Bactericidal Concentrations (MIC and MBC)

The minimum concentration capable of inhibiting the growth of the bacteria or fungi (MIC) was determined by a modified microdilution method [46]. Cinnamaldehyde (2000, 1000, 500, 250, 125, 62.5, 31.25, and 6.625 µg/mL final well concentration) and alpha terpineol (5000, 2500, 1250, 625, 312.5, 156.25, 78.125, and 39.0625 µg/mL final well concentration) were prepared in tryptic soy broth (TSB). Experiments were performed in 96-well microplates. The bacterial suspension (0.5 McFarland with 10^8^ CFU/mL) was diluted to 10^7^ CFU/mL with sterile TSB. The yeast suspension was standardized at 0.5 McFarland with 10^6^ CFU/mL. The diluted compounds were added to the wells at 180 µL. Then, 20 µL of bacterial and yeast suspensions were added, resulting in a final concentration of 10^6^ CFU/mL for bacteria and 10^5^ CFU/mL for yeast. The plates were incubated at 37 °C for 24 h for bacteria and 30 °C for 48 h for yeast, and the MIC was determined by the concentration where no visible growth of the bacteria or yeast was observed. The growth control for the experiment was a bacterial suspension in TSB with no external compound. For the minimum bactericidal concentration (MBC), the concentrations that had no apparent growth on the MIC test were spread on chocolate agar plates (Remel, USA) for bacteria and SDA agar for fungi and incubated at 37 °C for 24 h for bacteria or at 30 °C for yeast for 48 h. Then, the incubated plates were inspected, and the lowest concentration with no visible growth was deemed the MBC.

### 3.8. Structural Morphological Changes of the Bacteria

For light microscope observation, a treated bacteria sample from the border of the zone of inhibition and the bacteria control were suspended in sterile distilled water on a microscope slide and heat-fixed. A Gram stain was performed and observed microscopically with an Olympus CX 23 video microscope.

### 3.9. Statistical Analysis

The data are shown as the mean of three independent replicates with standard deviation. One-way analysis of variance was used for comparison (α = 5% of significance) to detect significant differences between the antioxidant activity of the cranberry samples using Origin Pro software 10.1.5.132. Origin and Microsoft Excel were used to generate graphs for visualization. The IC_25_ and IC_30_ values were determined by the non-linear regression method using Excel. Multivariate hierarchical cluster analysis was used to determine the pattern of inhibition of the bacteria and fungi by the cranberry samples and compounds (Origin Pro software). The input variable data were the mean zones of inhibition of the compounds. The cluster method was based on the furthest neighbor, and the distance was calculated by Euclidean type. The Y-axis was reported as similarity among the clusters.

## 4. Conclusions

In this study, we report a first attempt to characterize the volatile profile of cranberry plant aerial parts prepared by utilizing hydrodistillation techniques. We also report the antibacterial and antifungal screening of the cranberry plant volatile extract for the first time. The EB and MQ vine volatile extracts varied in the DPPH inhibition activity among the ST and CM techniques, with the former technique demonstrating better activity than the latter. Future research should focus on the differences in the antioxidant behavior of the cranberry vine under different extraction techniques. Also, the specific compounds contributing to the antioxidant behavior of the Clevenger extracts should be scrutinized further. Moreover, we report selective antibacterial and antifungal activities of the Clevenger leaf and vine volatiles based on the disc diffusion assay.

Future research should focus on the screening of individual compounds that could account for the antimicrobial behavior of the Clevenger extracts. The major constituent in the leaf by the steam distillation method, trans-cinnamaldehyde, demonstrated excellent antibacterial and antifungal behavior. This suggests that synergistic activity could account for the weak antimicrobial behavior of the steam-distilled volatile extract. Preliminary observation by light microscopy revealed an obvious effect of trans-cinnamaldehyde on the bacterial cell morphology of *E. coli* and a more subtle effect on *S. aureus*. Based on our results, we propose that cranberry plant volatile extracts could be promising potential antioxidants with antibacterial and antifungal activities.

## Figures and Tables

**Table 1:** Chemical composition of cranberry leaf volatiles reported in percentage area ± standard deviation.

RT	Compound Identified	Steam Distillation			Clevenger Hydrodistillation			Molecular Weight
		EB	MQ	ST ^++^	EB	MQ	ST	
4.78	Hexanal	1.89	0.79	N/A	N/A	N/A	N/A	100
5.69	3-Furaldehyde	2.69	N/A	N/A	N/A	N/A	N/A	96
7.19	Styrene	0.61	0.75	N/A	N/A	N/A	N/A	104
7.49	Heptanal	0.29	0.43	N/A	N/A	N/A	N/A	114
9.33	Benzaldehyde	1.87	N/A	N/A	N/A	N/A	N/A	106
9.47	5-methylfurfural	3.44	0.58	N/A	N/A	N/A	N/A	110
10.57	Octanal	1.88	2.48	N/A	N/A	N/A	N/A	128
11.23	O-cymene	N/A	N/A	N/A	N/A	N/A	N/A	152
11.32	Benzeneethanol, β-ethenyl-α-methyl-	1.59	N/A	N/A	N/A	N/A	N/A	162
11.78	Isophorone	0.34	0.46	N/A	N/A	N/A	N/A	138
11.94	Benzyl alcohol	N/A	N/A	N/A	N/A	1.27	N/A	108
11.96	Unknown	1.33	N/A	N/A	N/A	N/A	N/A	150
12.57	Ethyl sorbate	N/A	N/A	N/A	1.08	1.45	N/A	140
12.74	Unknown	1.80	1.21	N/A	N/A	N/A	N/A	152
12.86	Unknown	N/A	N/A	N/A	N/A	N/A	1.52	97
12.97	Unknown	1.01	N/A	N/A	N/A	N/A	N/A	152
**13.03**	**7-oxanorbornene**	**N/A**	**N/A**	**N/A**	**3.86**	**51.4**	**Trace**	**96**
13.28	4-Vinyl-o-xylene	0.39	0.21	N/A	N/A	N/A	N/A	132
13.61	Cis Geraniol	0.47	0.44	N/A	N/A	N/A	N/A	154
13.75	Nonanal	1.72	3.23	N/A	N/A	N/A	N/A	142
15.09	Ketoisophorone	0.26	0.28	N/A	N/A	N/A	N/A	170
15.67	Unknown	1.44	1.46	N/A	N/A	N/A	N/A	136
15.83	Unknown	1.59	N/A	N/A	N/A	N/A	N/A	150
16.20	Dodecene	N/A	N/A	N/A	0.33	N/A	1.07	168
**16.33**	**alpha terpineol**	**4.97**	**16.4**	**N/A**	**N/A**	**N/A**	**N/A**	**154**
16.88	Dodecane	N/A	N/A	N/A	1.77	N/A	N/A	170
17.03	Cyclocitral	0.57	0.61	N/A	N/A	N/A	N/A	152
**17.35**	**Benzenepropanal**	**14.2**	**17.8**	**N/A**	**0.24**	**2.15**	**N/A**	**136**
**17.83**	**2-Decenal**	**6.13**	**5.25**	**N/A**	**N/A**	**N/A**	**N/A**	**154**
**18.14**	**Cinnamaldehyde (E)**	**12.8**	**27.8**	**N/A**	**N/A**	**N/A**	**N/A**	**132**
18.24	Unknown	N/A	N/A	N/A	0.57	2.09	N/A	210
18.90	Unknown	6.60	3.62	N/A	N/A	N/A	N/A	150
18.90	Tridecene	N/A	N/A	N/A	3.36	2.70	N/A	182
19.34	Ethyl phenethyl ketone	N/A	N/A	N/A	0.53	1.76	N/A	134
19.42	Longipinane	0.59	0.83	N/A	N/A	N/A	N/A	206
19.65	Unknown	1.20	N/A	N/A	N/A	N/A	N/A	250
19.87	1,1-Diethoxynonae	N/A	N/A	N/A	N/A	N/A	0.25	216
19.99	5-methylheptanol	N/A	N/A	N/A	N/A	N/A	0.48	130
20.03	Tetradecene	N/A	N/A	N/A	0.71	1.01	N/A	196
20.11	Methoxyacetic acid	N/A	N/A	N/A	N/A	N/A	0.68	90
20.97	Geranyl acetone	N/A	N/A	N/A	0.57	0.94	Trace	194
21.00	Unknown	1.11	0.60	N/A	N/A	N/A	N/A	244
21.22	Unknown	2.18	0.94	N/A	N/A	N/A	N/A	190
21.31	Pentadecane	N/A	N/A	N/A	N/A	0.66	N/A	212
21.53	beta Ionone	1.09	1.41	N/A	N/A	N/A	N/A	192
21.54	Germacrene d	N/A	N/A	N/A	N/A	0.58	Trace	204
21.61	Ionone	N/A	N/A	N/A	0.35	N/A	0.33	192
21.92	1-(2,3,6-trimethylphenyl-3-buten-2-one	N/A	N/A	N/A	N/A	1.13	N/A	188
21.92	1-(2,3,6-trimethylphenyl-3-buten-2-one	5.53	0.88	N/A	N/A	N/A	N/A	188
22.25	Pentadecene	N/A	N/A	N/A	N/A	1.99	1.36	210
22.39	Unknown	2.12	0.45	N/A	N/A	N/A	N/A	188
22.51	Dodecanoic acid	N/A	N/A	N/A	0.84	N/A	1.44	200
22.58	Unknown	1.15	N/A	N/A	N/A	N/A	N/A	278
22.75	Hexadecene	N/A	N/A	N/A	0.73	2.93	1.27	224
22.81	Megastigmatrienone	3.06	0.62	N/A	N/A	N/A	N/A	190
23.41	Megastigmatrienone (Isomer)	2.37	0.36	N/A	N/A	N/A	N/A	190
23.49	Unknown	N/A	N/A	N/A	0.87	N/A	1.33	356
23.79	Hexadecane	N/A	N/A	N/A	0.96	2.18	0.59	226
23.86	Eudesm-7(11)-en-4-ol	1.57	0.27	N/A	N/A	N/A	N/A	222
24.01	Heptadecane	N/A	N/A	N/A	2.91	1.06	Trace	240
24.05	Unknown	N/A	3.46	N/A	N/A	N/A	N/A	250
24.59	Tetradecanol	N/A	N/A	N/A	1.79	0.28	1.62	214
24.67	Pentadecanol	N/A	N/A	N/A	N/A	0.84	1.62	228
24.79	Tetradecanoic acid	N/A	N/A	N/A	N/A	N/A	10.8	228
24.87	Heptadecene	N/A	N/A	N/A	2.09	2.28	N/A	238
25.04	Octadecene	N/A	N/A	N/A	0.52	1.75	1.54	252
25.15	Unknown	2.22	1.40	N/A	N/A	N/A	N/A	278
**25.49**	**Phytol (isomer)**	**N/A**	**N/A**	**N/A**	**3.76**	**1.14**	**13.8**	**296**
25.87	2-Methyl-7-octadecyne	N/A	N/A	N/A	0.95	N/A	2.77	264
26.00	2-Methyl-7-octadecyne (isomer)	N/A	N/A	N/A	1.57	0.55	4.99	264
**26.45**	**Methyl palmitate**	**N/A**	**N/A**	**N/A**	**2.88**	**4.20**	**1.76**	**270**
26.85	Hexadecanoic acid	N/A	N/A	N/A	4.44	N/A	4.54	256
27.07	Unknown	N/A	N/A	N/A	N/A	N/A	2.38	286
27.10	Heptadecanoic acid	N/A	N/A	N/A	2.03	2.64	N/A	282
27.45	Unknown	0.40	N/A	N/A	N/A	N/A	N/A	286
27.51	Kaur-15-ene	N/A	N/A	N/A	N/A	0.56	N/A	272
27.95	Octadecanol	N/A	N/A	N/A	5.58	N/A	5.06	270
27.96	Kaur-15-ene (isomer)	0.30	0.35	N/A	N/A	N/A	N/A	272
**27.99**	**Kaur-16-ene**	**3.49**	**0.62**	**N/A**	**6.27**	**N/A**	**5.06**	**272**
28.14	Methyl Linolenate	N/A	N/A	N/A	2.41	N/A	0.70	292
**28.28**	**Phytol**	**1.79**	**4.00**	**N/A**	**8.76**	**2.89**	**6.70**	**296**
28.45	Heniecosane	N/A	N/A	N/A	N/A	0.93	N/A	296
28.58	Linoleic acid	N/A	N/A	N/A	N/A	0.93	N/A	280
28.78	Linoleic acid (isomer)	N/A	N/A	N/A	2.42	2.58	N/A	280
28.95	Docosane	N/A	N/A	N/A	1.49	1.30	20.9	380
29.38	Unknown	N/A	N/A	N/A	N/A	1.17	3.44	306
**29.55**	**Methyl strictate**	**N/A**	**N/A**	**N/A**	**32.3**	**N/A**	**Trace**	**328**
29.87	Unknown	N/A	N/A	N/A	1.10	0.67	2.19	280
	**Total % Area**	**100.0**	**100.0**	**N/A**	**100.0**	**100.0**	**100.0**	

^++^ Steam distillation was not performed for Stevens leaf cultivar due to unavailability of sample. N/A means compound absent. Compounds with 0.20% area and above were considered. Trace (0.05–0.1%).

**Table 2:** Chemical composition of cranberry vine volatiles reported in percentage area ± standard deviation.

RT	Compounds	Steam Distillation			Clevenger Hydrodistillation			Molecular Weight
		EB	MQ	ST ^++^	EB	MQ	ST	
4.13	Acetone, diethyl acetal	N/A	N/A	N/A	N/A	0.55	N/A	132
4.79	Hexanal	1.28	1.85	N/A	N/A	N/A	N/A	100
6.55	p-Xylene	Trace	0.33	N/A	N/A	N/A	N/A	106
7.20	Styrene	Trace	0.70	N/A	N/A	N/A	N/A	227
9.27	Unknown	0.85	Trace	N/A	N/A	N/A	N/A	164
10.20	Unknown	0.96	0.82	N/A	N/A	N/A	N/A	138
10.98	Unknown	Trace	0.22	N/A	N/A	N/A	N/A	136
11.36	Unknown	Trace	0.74	N/A	N/A	N/A	N/A	136
12.31	Unknown	Trace	0.89	N/A	N/A	N/A	N/A	138
12.76	Trans-p-mentha-2,8-dienol	0.26	0.87	N/A	N/A	N/A	N/A	152
12.92	Furfural diethyl acetal	N/A	N/A	N/A	N/A	0.91	N/A	170
13.23	1,5,5-trimethyl-6-methylenecyclohexene	Trace	0.82	N/A	N/A	N/A	N/A	136
13.62	Citrylidene ethanol	Trace	2.11	N/A	N/A	N/A	N/A	182
13.78	Nonanal	1.78	2.81	N/A	N/A	N/A	N/A	142
13.95	2-Methylcumarone	Trace	1.51	N/A	N/A	N/A	N/A	124
14.94	trans Sabinol	3.36	1.08	N/A	N/A	N/A	N/A	152
15.72	Borneol	0.98	1.46	N/A	N/A	N/A	N/A	154
16.00	Terpinen-4-ol	1.81	2.69	N/A	N/A	N/A	N/A	154
16.24	Dodecene	N/A	N/A	N/A	1.05	0.39	0.86	168
**16.34**	**alpha terpineol**	**8.39**	**25.2**	**N/A**	**N/A**	**N/A**	**N/A**	**154**
**16.49**	**Myrtenol**	**2.27**	**9.28**	**N/A**	**N/A**	**N/A**	**N/A**	**152**
16.63	Unknown	1.19	N/A	N/A	N/A	N/A	N/A	150
17.03	Unknown	0.76	1.13	N/A	N/A	N/A	N/A	100
17.81	2-Furancarboxaldehyde	N/A	N/A	N/A	Trace	Trace	1.98	96
17.99	Unknown	2.49	2.59	N/A	N/A	N/A	N/A	150
18.26	p-Ethylguaicol	N/A	6.64	N/A	N/A	N/A	N/A	152
18.61	2-Tridecene-E	N/A	N/A	N/A	0.30	0.50	Trace	182
18.75	Unknown	0.88	Trace	N/A	N/A	N/A	N/A	256
18.76	2-Tridecene-E (isomer)	N/A	N/A	N/A	0.51	0.69	N/A	182
18.90	Tridecene (isomer)	N/A	N/A	N/A	0.38	0.69	Trace	182
19.31	Unknown	1.13	0.75	N/A	N/A	N/A	N/A	150
19.67	Unknown	0.74	1.42	N/A	N/A	N/A	N/A	224
20.02	Tetradecene	N/A	N/A	N/A	1.95	1.16	3.24	196
21.16	m-Eugenol	Trace	3.81	N/A	N/A	N/A	N/A	164
21.55	γ-Selinene	1.89	3.26	N/A	0.64	0.89	1.26	204
21.86	Unknown	0.60	2.85	N/A	N/A	N/A	N/A	266
21.87	Phenol, 2,4-ditertbutyl	N/A	N/A	N/A	2.78	1.46	Trace	206
22.02	Delta Amorphene	N/A	N/A	N/A	0.67	1.15	0.39	204
22.25	Unknown	N/A	N/A	N/A	2.09	1.25	0.60	256
22.74	Tridecanol	N/A	N/A	N/A	3.07	0.88	3.34	200
**23.86**	**Eudesm-7(11)-en-4-ol**	**49.8**	**22.9**	**N/A**	**3.33**	**1.20**	**2.63**	**222**
24.23	Unknown	N/A	N/A	N/A	N/A	1.53	N/A	268
24.58	Unknown	N/A	N/A	N/A	N/A	1.93	N/A	306
24.79	Unknown	N/A	N/A	N/A	5.29	1.53	N/A	306
25.03	Octadecene	N/A	N/A	N/A	3.83	2.42	1.88	252
25.63	Unknown	N/A	N/A	N/A	1.34	N/A	N/A	322
26.22	Unknown	N/A	N/A	N/A	1.58	1.57	N/A	344
27.00	Hexadecanoic acid	N/A	N/A	N/A	Trace	0.34	12.8	256
**27.10**	**Eicosene**	**N/A**	**N/A**	**N/A**	**6.25**	**5.82**	**2.72**	**280**
**27.44**	**Methyl linoleate**	**N/A**	**N/A**	**N/A**	**16.6**	**2.34**	**11.3**	**294**
**28.10**	**Heneicosane**	**N/A**	**N/A**	**N/A**	**1.63**	**1.64**	**2.28**	**296**
28.28	Phytol	N/A	N/A	N/A	2.04	1.58	0.40	296
28.56	Unknown	N/A	N/A	N/A	0.88	0.77	12.4	298
28.98	Docosene	N/A	N/A	N/A	19.3	3.17	3.05	308
29.17	Unknown	7.99	Trace	N/A	N/A	N/A	N/A	294
**29.62**	**Labd-(13E)-8,15-diol**	**N/A**	**N/A**	**N/A**	**23.9**	**63.6**	**36.8**	**306**
29.66	Unknown	10.6	1.37	N/A	N/A	N/A	N/A	280
29.94	Tetracosane	N/A	N/A	N/A	0.57	Trace	1.99	338
	**Total % Area**	**100.0**	**100.0**	**N/A**	**100.0**	**100.0**	**100.0**	

^++^ Steam distillation was not performed for Stevens cultivar vine due to unavailability of sample. N/A means compound absent. Compounds with 0.20% area and above were considered. Trace (0.05–0.1%).

**Table 3:** Antibacterial activity of cranberry leaf and vine volatile extracts prepared using steam distillation (SD) and Clevenger method (CM) by disc diffusion; mean zone of inhibition in mm.

	*B. cereus*	*S. aureus*	*E. coli*	*P. aeruginosa*	*C. albicans*
					
EB leaf (SD)	8.00	7.00	7.00	6.00	8.00
MQ leaf (SD)	6.00	7.00	6.00	6.00	7.00
					
EB vine (SD)	7.00	6.00	7.00	6.00	6.00
MQ vine (SD)	8.00	6.00	6.00	6.00	6.00
					
EB leaf (CM)	15.33	14.67	11.33	7.67	32.33
MQ leaf (CM)	14.33	12.33	14.33	8.00	30.00
ST leaf (CM)	11.33	11.67	6.00	6.00	6.00
EB vine (CM)	8.67	10.33	6.00	9.33	13.33
MQ vine (CM)	7.67	8.67	6.00	7.00	19.33
ST vine (CM)	7.33	7.33	6.00	6.00	11.67
*Positive controls* Erythromycin	33.67	30.00	N/A	N/A	N/A
Tobramycin	N/A	N/A	22.00	24.33	N/A
Miconazole nitrate	N/A	N/A	N/A	N/A	10.00

Steam distillation was not performed for ST leaf and vine; therefore, no screening was possible. N/A = not applicable. Mean zone of inhibition of 6 mm = no activity.

**Table 4:** Minimum inhibitory concentration (MIC) and minimum bactericidal concentration (MBC) of *E*. *coli*, *S. aureus*, *B. cereus*, *P. aeruginosa,* and *C. albicans*.

Organism	Cinnamaldehyde (mg/mL)	Cinnamaldehyde (Literature) (mg/mL) [59,62]	Alpha Terpineol (mg/mL)	Alpha Terpineol (Literature) (mg/mL) [59]
*B. cereus* (MIC)	0.250	0.031	5	N/A
*B. cereus* (MBC)	0.250	N/A	5	N/A
*S. aureus* (MIC)	0.250	0.62	5	0.747
*S. aureus* (MBC)	1.000	5	5	1.868
*E. coli* (MIC)	0.500	1.25	2.5	0.747
*E. coli* (MBC)	0.500	5	2.5	1.868
*P. aeruginosa* (MIC)	0.667	0.31	>5	N/A
*P. aeruginosa* (MBC)	0.500	2.5	>5	N/A
*C. albicans* (MIC)	0.250	0.08	2.5	N/A
*C. albicans* (MFC)	0.250	0.31	2.5	N/A

N/A means value not found in the literature.

## Data Availability

Data are contained within the article.

## Supplementary Materials

The following supporting information can be downloaded at: https://www.mdpi.com/article/10.3390/molecules30092047/s1, Table S1. Identification of cranberry leaf compounds by C7-C40 alkane standard mixture; Table S2. Identification of cranberry vine compounds by C7-C40 alkane standard mixture; Figure S1. GC-MS chromatogram of alkane standard mixture for calculation of linear retention index and identification of compounds; Figure S2. Representative GC-MS chromatogram of steam distillation cranberry vine (MQ cultivar); Figure S3. Representative GC-MS chromatogram of steam distillation cranberry leaf (MQ cultivar); Figure S4. Representative GC-MS chromatogram of modified Clevenger volatile cranberry leaf (MQ cultivar); Figure S5. Representative GC-MS chromatogram of modified Clevenger volatile cranberry vine (MQ cultivar); Figure S6. Disc diffusion assay of A-terpineol against tested bacterial organisms *B. cereus* ATCC 11778; *E. coli* ATCC 25922; *S. aureus* ATCC 25923; and *P. aeruginosa* ATCC 27853; Figure S7. Disc diffusion assay of cranberry vine against tested bacterial organisms *B. cereus* ATCC 11778; *E. coli* ATCC 25922; *S. aureus* ATCC 25923; and *P. aeruginosa* ATCC 27853; Figure S8. Optical density (600 nm) of trans-cinnamaldehyde MIC against tested bacterial organisms. Data represent the means of triplicate analysis; Figure S9. Optical density (600 nm) of trans-cinnamaldehyde and A-Terpineol MIC against *C. albicans*. Data represent the means of triplicate analysis; Figure S10. Optical density (600 nm) of α-terpineol against tested bacterial organisms. Data represent the means of triplicate analysis.
