# Supplementary material for: Gas Chromatography/Mass Spectrometry Chemical Profiling of Volatile Compounds from Cranberry Plant Byproducts as Potential Antibacterials, Antifungals, and Antioxidants
Source: Molecules. 2025 May 4;30(9):2047. doi: 10.3390/molecules30092047 (PMC12074010; doi:10.3390/molecules30092047)
Supplement: Supplementary file 1 [file molecules-30-02047-s001.zip › molecules-3565001-supplementary.pdf]

molecules

Supplementary

Gas Chromatography/Mass spectrometry chemical profiling of volatile compounds from cranberry plant byproducts as potential antibacterials, antifungals and antioxidants

Martin Aborah,[1] **Frank Scarano,**[2] **and Catherine Neto** [1, *]

[1] University of Massachusetts Dartmouth, Department of Chemistry and Biochemistry; maborah@umassd.edu
[2] University of Massachusetts Dartmouth, Medical Laboratory Science Department; fscarano@umassd.edu
* Correspondence: cneto@umassd.edu; Tel.: +1 508-910-6654

Citation: To be added by editorial staff during production.

Academic Editor: Firstname Lastname

Received: date
Revised: date
Accepted: date
Published: date

Copyright: © 2024 by the authors. Submitted for possible open access publication under the terms and conditions of the Creative Commons Attribution (CC BY) license (https://creativecommons.org/licenses/by/4.0/).

Table S1. Identification of cranberry leaf compounds by C7-C40 alkane standard mixture

	Clevenger hydrodistillation				Steam distillation		
**RT**	**Cranberry leaf compounds**	**Arithmetic index (Calculated)**	**Arithmetic index (literature)***	**RT**	**Cranberry leaf compounds**	**Arithmetic index (Calculated)**	**Arithmetic index (literature)***
11.94	*Benzyl alcohol*	*1044*	*1035*	*4.78*	*Hexanal*	*800*	*801*[1]
12.57	*Ethyl sorbate*	*1065*	*1071*	*5.69*	*3-Furaldehyde*	*834*	*831*[2]
12.86	*Unknown*	*1073*	*N/A*	*7.19*	*Styrene*	*890*	*893*[3]
13.03	*7-oxanorbornenone*	*1079*	*N/A*	*7.49*	*Heptanal*	*901*	*903*[4]
16.20	*Dodecene*	*1188*	*1187*	*8.49*	*Unknown*	*933*	*N/A*
16.88	*Dodecane*	*1216*	*1200*	*9.33*	*Benzaldehyde*	*960*	*960*[5]
17.36	*Benzenepropanal*	*1240*	*1231*	*9.47*	*5-methylfurfural*	*965*	*978*[6]
18.24	*Unknown*	*1283*	*N/A*	*10.57*	*Octanal*	*1000*	*998*[7]
18.90	*Tridecene*	*1319*	*1293*	*11.78*	*Isophorone*	*1039*	*1044*[8]
19.34	*Ethyl phenethyl ketone*	*1346*	*1336*	*11.23*	*O-cymene*	*1022*	*1022*[9]
19.87	*1,1-Diethoxynonae*	*1378*	*1374*	*11.32*	*Benzeneethanol, β-ethenyl-α-methyl-*	*1025*	*N/A*
19.99	*5-methylheptanol*	*1385*	*N/A*	*11.97*	*Unknown*	*1045*	*N/A*
20.03	*Tetradecene*	*1389*	*1388*	*12.74*	*Unknown*	*1070*	*N/A*
20.11	*Methoxyacetic acid*	*1393*	*N/A*	*13.28*	*4-Vinyl-o-xylene*	*1087*	*1099*[10]
20.97	*Geranyl acetone*	*1452*	*1452*	*13.61*	*Cis Geraniol*	*1098*	*1225*[11]
21.31	*Pentadecane*	*1476*	*1500*	*13.75*	*Nonanal*	*1102*	*1104*[12]
21.54	*Germacrene d*	*1492*	*1491*	*14.93*	*Unknown*	*1144*	*N/A*
21.61	*Ionone*	*1497*	*1487*	*15.09*	*Ketoisophorone*	*1149*	*1147*[13]

1 *10.1021/acs.jafc.6b01150*

2 *https://doi.org/10.5194/acp-3-665-2003. Retrieved from NIST.*

3 *http://flavornet.org/flavornet.html*

4 *http://flavornet.org/flavornet.html*

5 *http://flavornet.org/flavornet.html*

6 *http://flavornet.org/flavornet.html*

7 *Adams, R. P. Identification of Essential Oil Components by Gas Chromatography Mass Spectroscopy; Allured Publishing Corporation: Carol Stream, Ill, 2007.*

8 *https://doi.org/10.1006/fstl.2001.0819. Retrieved from NIST*

9 *https://doi.org/10.1016/j.bse.2006.09.015. Retrieved from NIST*

10 *https://dx.doi.org/10.1016/S0021-9673(01)97947-6. Retrieved from NIST.*

11 *https://doi.org/10.1021/jf070997q. Retrieved from NIST.*

12 *Food Funct., 2019, 10, 7091–7102*

13 *Lalel, H.J.D.; Singh, Z.; Chye Tan, S., Glycosidically-bound aroma volatile compounds in the skin and pulp of 'Kensington Pride' mango fruit at different stages of maturity, Postharvest Biol. Technol., 29, 2003, 205-218*

21.92	*1-(2,3,6-trimethylphenyl-3-buten-2-one*	*1517*	*N/A*	*15.67*	*Unknown*	*1170*	*N/A*
22.25	*Pentadecene*	*1547*	*1502*	*16.33*	*alpha terpineol*	*1192*	*1195*[14]
22.51	*Dodecanoic acid*	*1567*	*1567*	*17.03*	*Cyclocitral*	*1224*	*1217*[15]
22.75	*Hexadecene*	*1586*	*1588*	*17.35*	*Benzenepropanal*	*1240*	*1231*[16]
23.49	*Unknown*	*1647*	*N/A*	*17.83*	*2-Decenal*	*1263*	*1260*[17]
23.79	*Hexadecane*	*1673*	*1600*	*18.14*	*Cinnamaldehyde (E)*	*1278*	*1283*[18]
24.01	*Heptadecane*	*1696*	*1700*	*18.90*	*Unknown*	*1319*	*N/A*
24.59	*Tetradecanol*	*1744*	*1686*	*19.42*	*Longipinane*	*1351*	*1350*[19]
24.67	*Pentadecanol*	*1751*	*1772*	*21.00*	*Unknown*	*1454*	*N/A*
24.79	*Tetradecanoic acid*	*1762*	*1771*	*21.22*	*Unknown*	*1469*	*N/A*
24.87	*Heptadecene*	*1770*	*1692*	*21.53*	*beta Ionone*	*1492*	*1493*[20]
25.04	*Octadecene*	*1785*	*1789*	*22.39*	*Unknown*	*1558*	*N/A*
25.49	*Phytol (isomer)*	*1828*	*N/A*	*22.81*	*Megastigma-trienone*	*1591*	*1582*[21]
25.87	*2-Methyl-7-octadecyne*	*1853*	*1863*	*23.41*	*Megastigma-trienone (Isomer)*	*1641*	*1656*[22]
26.00	*2-Methyl-7-octadecyne*	*1873*	*1863*	*23.86*	*Eudesm-7(11)-en-4-ol*	*1679*	*1682*[23]
26.45	*Methyl palmitate*	*1917*	*1921*	*25.15*	*Unknown*	*1795*	*N/A*
26.85	*Hexadecanoic acid*	*1963*	*1959*	*27.99*	*Kaur-16-ene*	*2079*	*2054*[24]
27.07	*Unknown*	*1986*	*N/A*	*28.28*	*Phytol*	*2124*	*2119*[25]
27.10	*Heptadecanoic acid*	*1989*	2038[26]	*28.95*	*Unknown*	*2287*	*N/A*

[14] *Food Funct., 2019, 10, 7091–7102*

[15] *Adams, R. P. Identification of Essential Oil Components by Gas Chromatography Mass Spectroscopy; Allured Publishing Corporation: Carol Stream, Ill, 2007.*

[16] *https://doi.org/10.1016/j.foodchem.2007.05.047. Retrieved from NIST.*

[17] *Adams, R. P. Identification of Essential Oil Components by Gas Chromatography Mass Spectroscopy; Allured Publishing Corporation: Carol Stream, Ill, 2007.*

[18] *http://flavornet.org/flavornet.html*

[19] *https://journals.sagepub.com/doi/pdf/10.1177/1934578X1501001151*

[20] *10.1021/acs.jafc.6b01150*

[21] *10.1007/s13197-015-2083-x*

[22] *10.1007/s13197-015-2083-x*

[23] *https://doi.org/10.1002/ffj.1330. Retrieved from NIST.*

[24] *https://doi.org/10.1016/j.indcrop.2011.04.016*

[25] *Todua, N.G., Retention Data. NIST Mass Spectrometry Data Center., NIST Mass Spectrometry Data Center, 2011*

[26] https://dx.doi.org/10.1021/jf00016a024

27.51	*Kaur-15-ene*	*2037*	*2036*	*N/A*	*N/A*	*N/A*	*N/A*
27.95	*Octadecanol*	*2077*	*2077*	*29.38*	*Unknown*	*2338*	*N/A*
27.98	*kaur-16-ene*	*2079*	*2061*	*29.55*	*Unknown*	*2355*	*N/A*
28.14	*Methyl Linolenate*	*2096*	*2095*	*29.87*	*Unknown*	*2387*	*N/A*
28.27	*Phytol*	*2124*	*2119*	*N/A*	*N/A*	*N/A*	*N/A*
28.45	*Heniecosane*	*2164*	*2100*	*N/A*	*N/A*	*N/A*	*N/A*
28.58	*Linoleic acid*	*2195*	*2132*	*N/A*	*N/A*	*N/A*	*N/A*
28.78	*Linoleic acid (isomer)*	*2245*	*2132*	*N/A*	*N/A*	*N/A*	*N/A*
28.95	*Docosane*	*2295*	*2300*	*N/A*	*N/A*	*N/A*	*N/A*
29.38	*Unknown*	*2338*	*N/A*	*N/A*	*N/A*	*N/A*	*N/A*
29.55	*Methyl strictate*	*2355*	*2387*	*N/A*	*N/A*	*N/A*	*N/A*

*Arithmetic index was obtained from the literature and published index in *Identification of essential oil components by gas chromatography mass spectroscopy* by Adams. *N/A* means index value absent.

Table S2. Identification of cranberry vine compounds by C7-C40 alkane standard mixture

	Clevenger hydrodistillation				Steam distillation		
**RT**	**Cranberry vine compounds**	**Arithmetic index (Calculated)**	**Arithmetic index (literature)**	**RT**	**Cranberry vine compounds**	**Arithmetic index (Calculated)**	**Arithmetic index (literature)**
4.13	*Acetone, diethyl acetal*	*763*	*761*	*4.79*	*Hexanal*	*800*	*801*[27]
12.92	*Furfural diethyl acetal*	*1076*	*1078*	*5.54*	*Unknown*	*828*	*N/A*
16.24	*Dodecene*	*1189*	*1187*	*6.55*	*p-Xylene*	*867*	*864*[28]
17.81	*2-Furancarboxaldehyde*	*1262*	*1267*	*7.20*	*Styrene*	*890*	*893*[29]
18.61	*2-Tridecene-E*	*1301*	*1293*	*9.27*	*Unknown*	*958*	*N/A*
18.76	*2-Tridecene-E (isomer)*	*1310*	*1315*	*10.20*	*Unknown*	*989*	*N/A*
18.90	*Tridecene (isomer)*	*1319*	*1315*	*10.98*	*Unknown*	*1014*	*N/A*
20.02	*Tetradecene*	*1389*	*1388*	*11.36*	*Unknown*	*1026*	*N/A*

[27] *10.1021/acs.jafc.6b01150*

[28] *https://dx.doi.org/10.1016/S0021-9673(02)00171-1. Retrieved from NIST.*

[29] *http://flavornet.org/flavornet.html*

21.52	*γ-Selinene*	*1491*	*1497*	*12.31*	*Unknown*	*1056*	*N/A*
21.87	*Phenol, 2,4-ditertbutyl*	*1512*	*1512*	*12.76*	*Trans-p-mentha-2,8-dienol*	*1071*	*N/A*
22.02	*Delta Amorphene*	*1529*	*1524*	*13.23*	*1,5,5-trimethyl-6-methylenecyclohexene*	*1086*	*N/A*
22.25	*Unknown*	*1547*	*N/A*	*13.62*	*Citrylidene ethanol*	*1098*	*N/A*
22.74	*Tridecanol*	*1585*	*1575*	*13.78*	*Nonanal*	*1102*	*1104*[30]
23.87	*Eudesm-7(11)-en-4-ol*	*1679*	*1682*	*13.95*	*2-Methylcumarone*	*1109*	*1109*[31]
24.23	*Unknown*	*1711*	*N/A*	*14.94*	*trans Sabinol*	*1144*	*1143*[32]
24.58	*Unknown*	*1743*	*N/A*	*15.72*	*Borneol*	*1171*	*1165*[33]
24.79	*Unknown*	*1762*	*N/A*	*16.00*	*Terpinen-4-ol*	*1181*	*1179*[34]
25.03	*Octadecene*	*1785*	*1789*	*16.34*	*alpha terpineol*	*1192*	*1195*[35]
25.63	*Unknown*	*1841*	*N/A*	*16.49*	*Myrtenol*	*1198*	*1198*[36]
26.22	*Unknown*	*1897*	*N/A*	*17.99*	*Unknown*	*1271*	*N/A*
27.00	*Hexadecanoic acid*	*1979*	*1959*	*18.26*	*p-Ethylguaicol*	*1284*	*1287*[37]
27.10	*Eicosene*	*1989*	*1987*	*19.67*	*Unknown*	*1366*	*N/A*
27.44	*Methyl linoleate*	*2024*	*2095*	*21.16*	*m-Eugenol*	*1465*	*1350*[38]
28.10	*Heneicosane*	*2091*	*2100*	*21.55*	*γ-Selinene*	*1492*	*1497*[39]
28.28	*Phytol*	*1586*	*1588*	*21.86*	*Unknown*	*1516*	*N/A*
28.56	*Unknown*	*2146*	*N/A*	*23.86*	*Eudesm-7(11)-en-4-ol*	*1679*	*1682*
28.60	*Unknown*	*2151*	*N/A*	*N/A*	*N/A*	*N/A*	*N/A*
28.98	*Docosene*	*2198*	*2195*	*N/A*	*N/A*	*N/A*	*N/A*
29.62	*Labd-(13E)-8,15-diol*	*2353*	*2422*	*N/A*	*N/A*	*N/A*	*N/A*
29.94	*Tetracosane*	*2394*	*2400*	*N/A*	*N/A*	*N/A*	*N/A*

[30] *Food Funct., 2019, 10, 7091–7102*

[31] *https://dx.doi.org/10.1002/jhrc.1240090603. Retrieved from NIST.*

[32] *https://doi.org/10.1002/ffj.1774. Retrieved from NIST.*

[33] *Adams, R. P. Identification of Essential Oil Components by Gas Chromatography Mass Spectroscopy; Allured Publishing Corporation: Carol Stream, Ill, 2007.*

[34] *Food Funct., 2019, 10, 7091–7102*

[35] *Food Funct., 2019, 10, 7091–7102*

[36] *https://doi.org/10.1002/ffj.1773. Retrieved from NIST.*

[37] *http://flavornet.org/flavornet.html*

[38] *https://dx.doi.org/10.1016/0031-9422(95)00241-X. Retrieved from NIST.*

[39] *https://doi.org/10.1002/ffj.1105. Retrieved from NIST.*

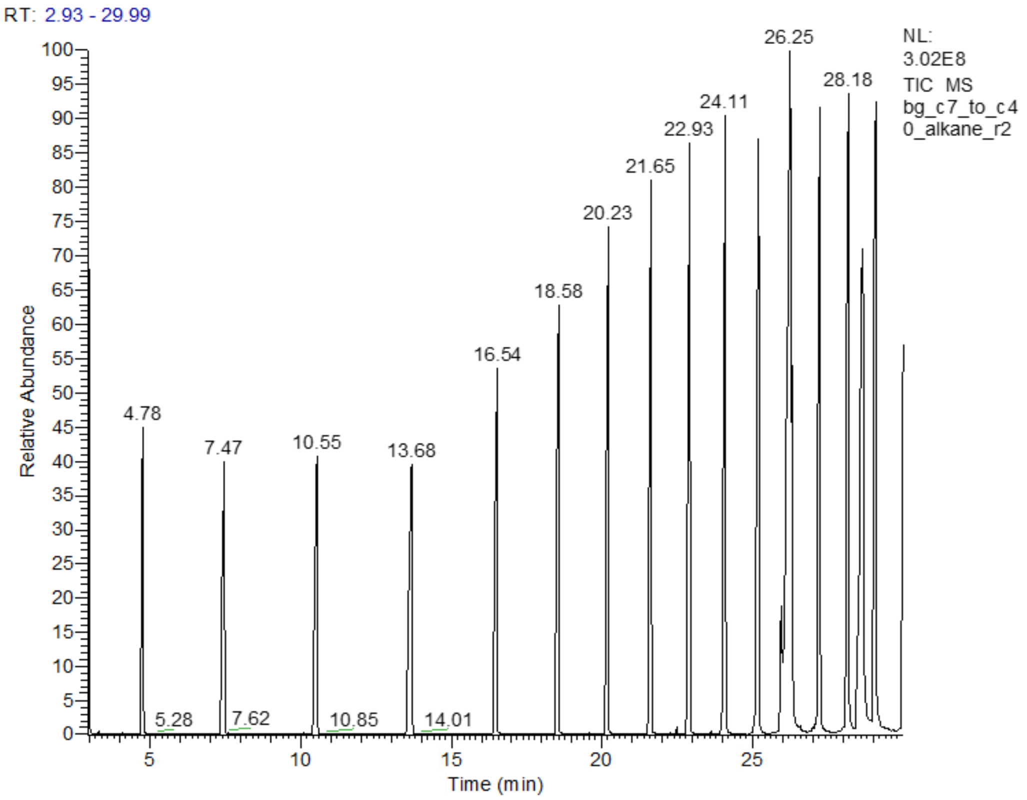

Figure S1. GCMS chromatogram of alkane standard mixture for calculation of linear retention index and identification of compounds.

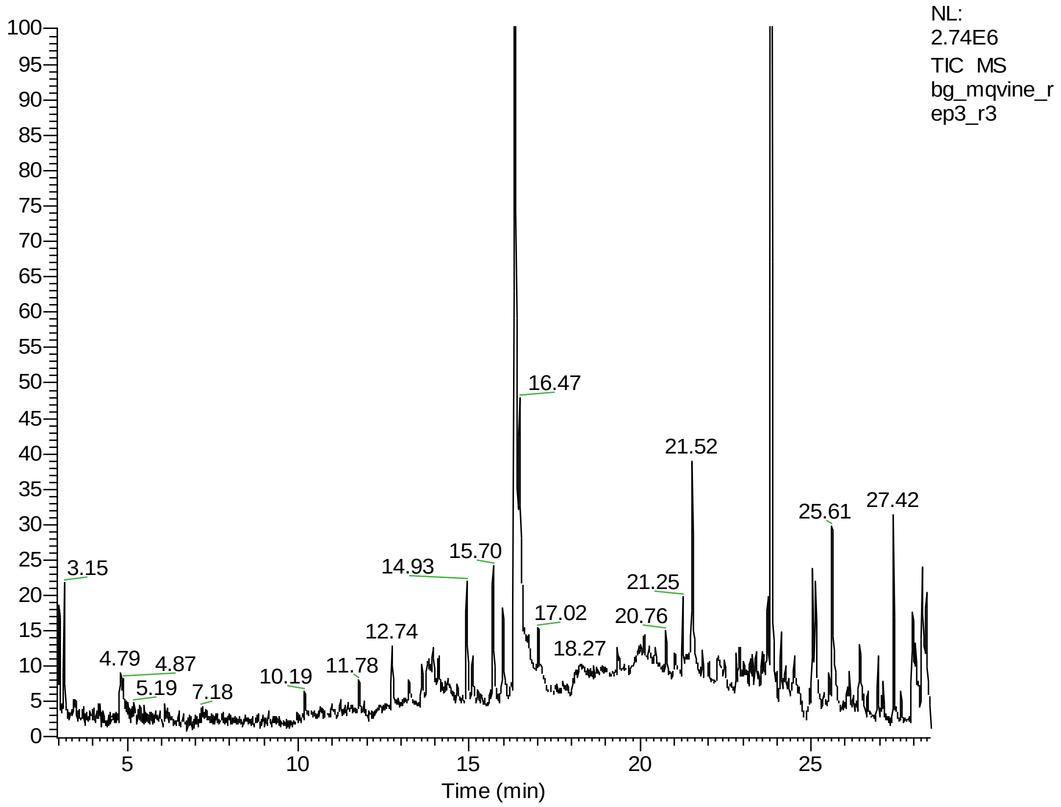

Figure S2. Representative GCMS chromatogram of steam distillation cranberry vine (MQ cultivar)

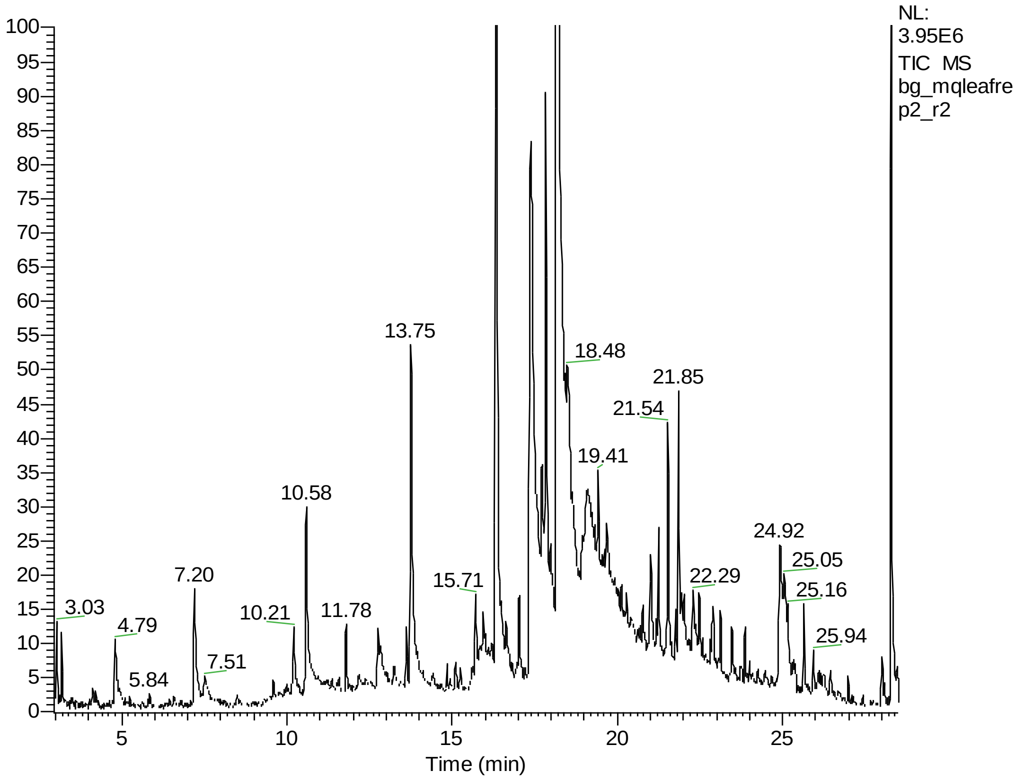

Figure S3. Representative GCMS chromatogram of steam distillation cranberry leaf (MQ cultivar).

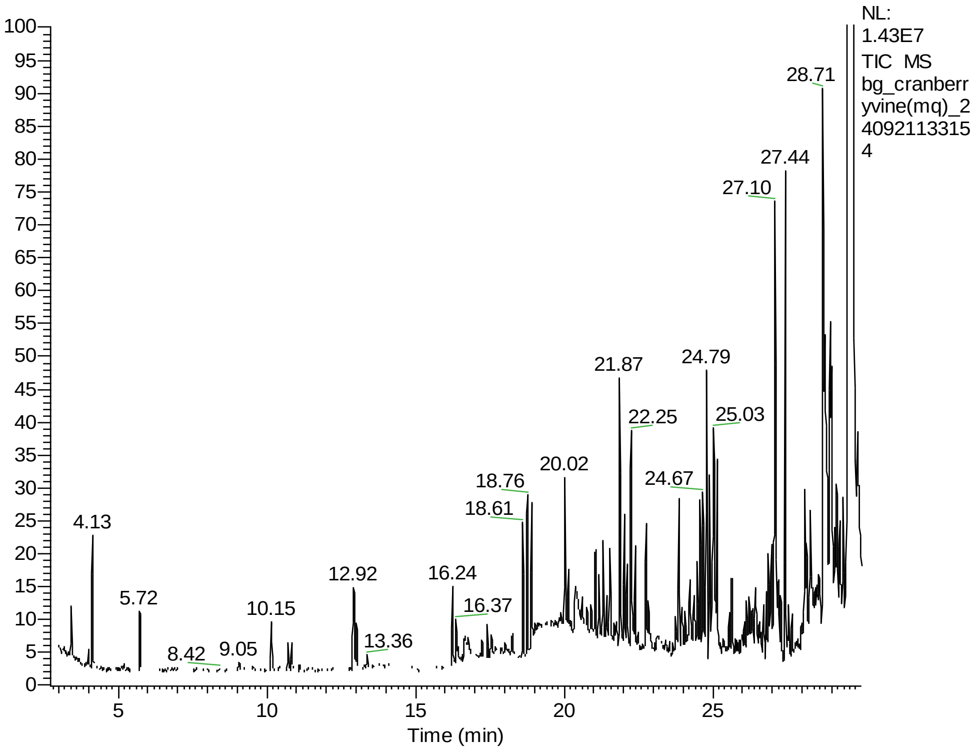

Figure S4. Representative GCMS chromatogram of modified Clevenger volatile cranberry leaf (MQ cultivar).

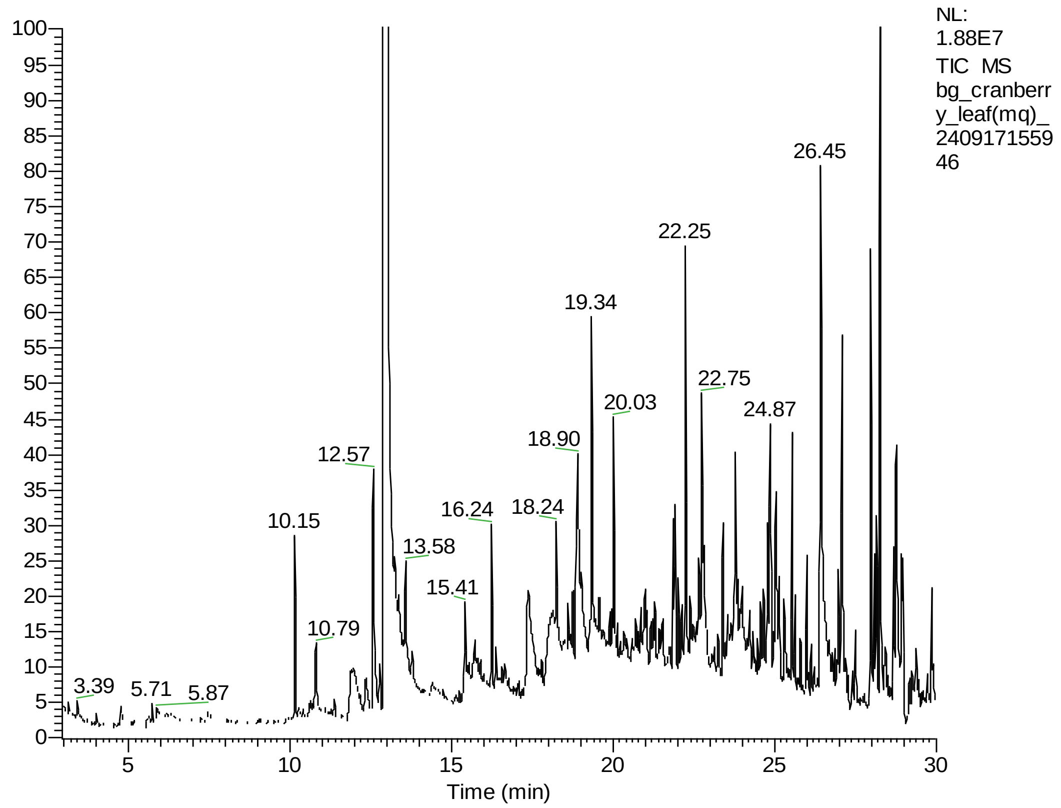

Figure S5. Representative GCMS chromatogram of modified Clevenger volatile cranberry vine (MQ cultivar).

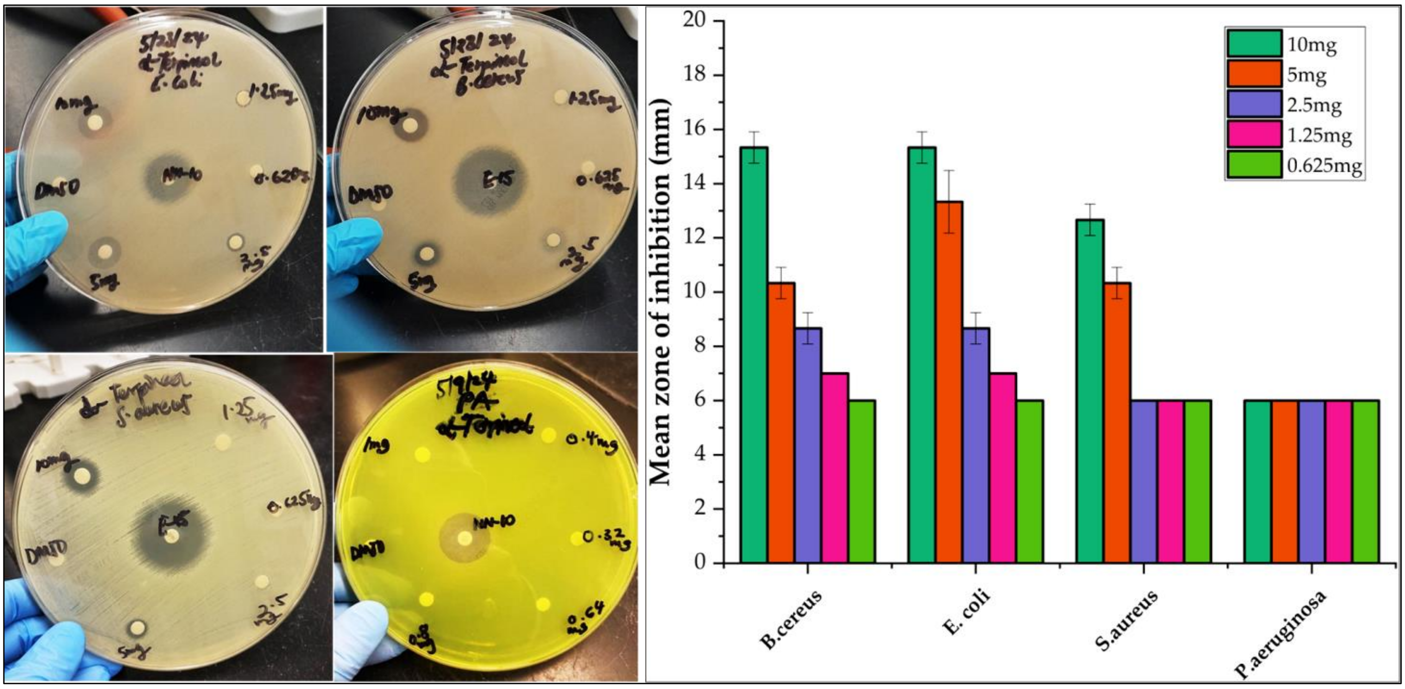

Figure S6. Disc diffusion assay of A-terpineol against test bacteria organisms. *B. cereus* ATCC 11778; *E. coli* ATCC 25922; *S. aureus* ATCC 25923; and *P. aeruginosa* ATCC 27853. NB: Mean zone of inhibition of 6 mm means no inhibition.

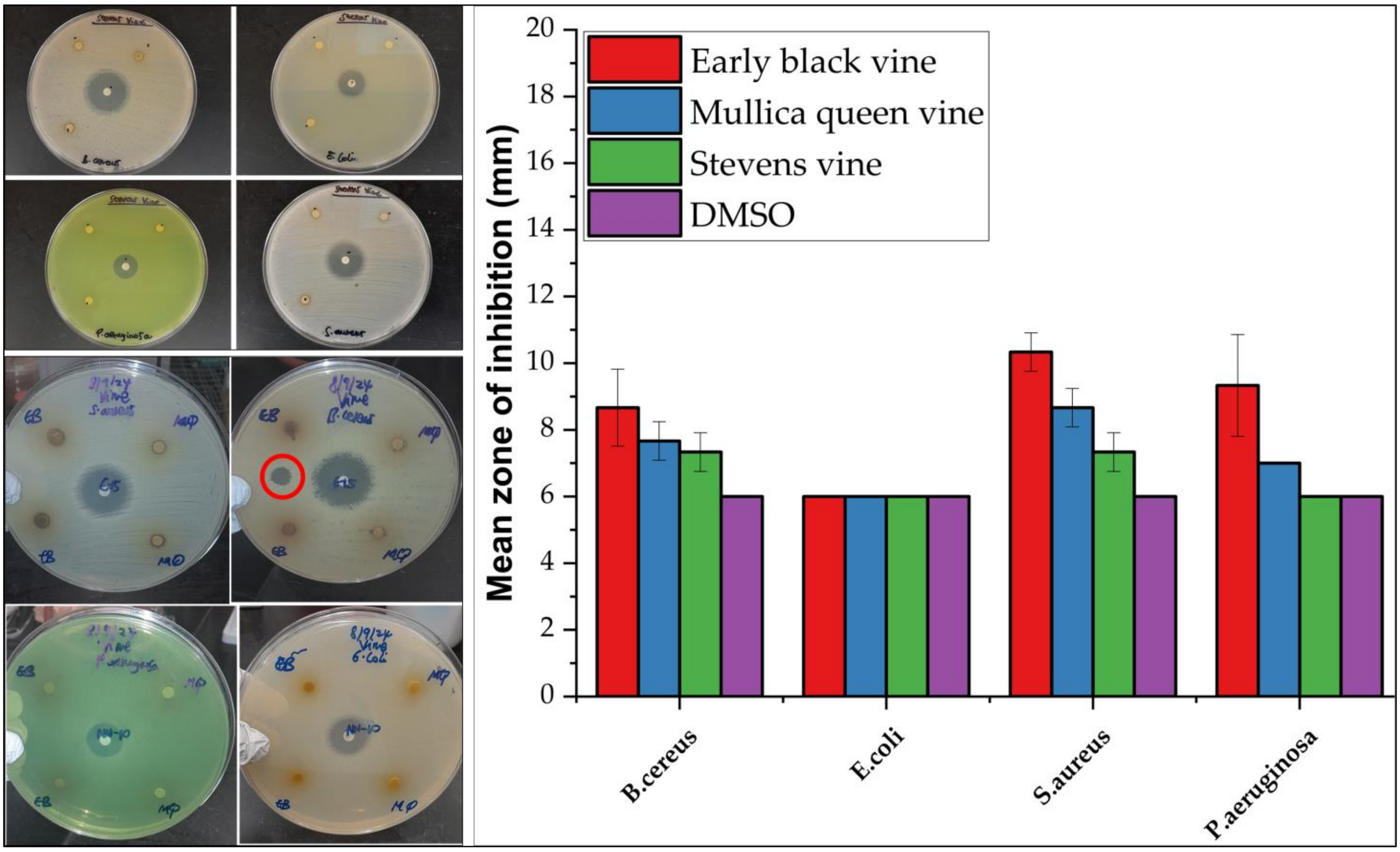

Figure S7. Disc diffusion assay of Cranberry vine against test bacteria organisms. Red circle indicates random error in the experiment. *B. cereus* ATCC 11778; *E. coli* ATCC 25922; *S. aureus* ATCC 25923; and *P. aeruginosa* ATCC 27853.

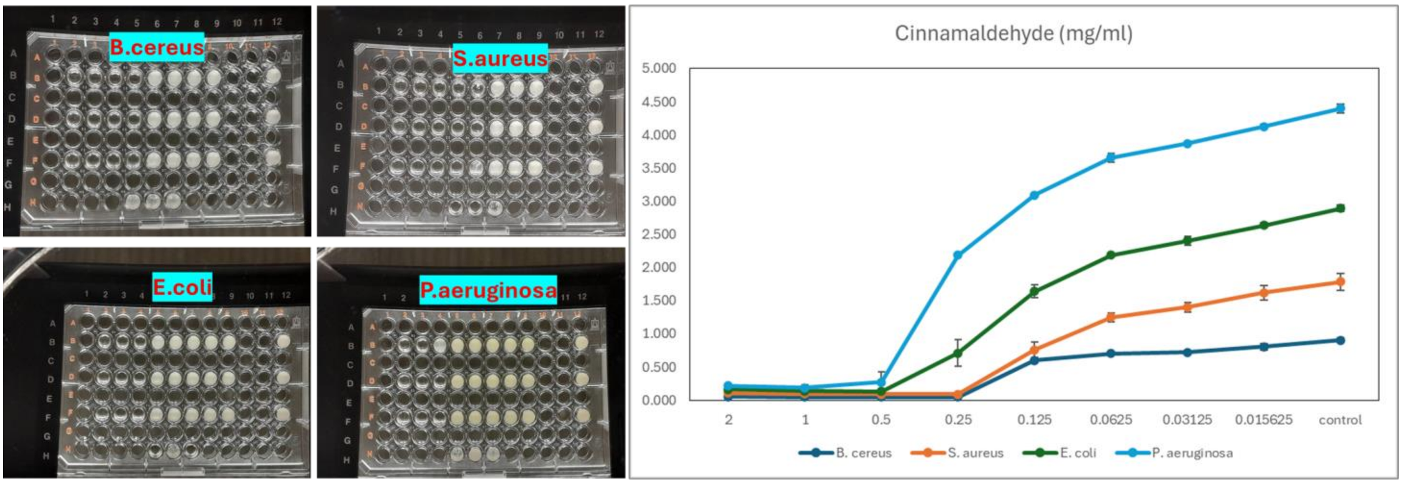

Figure S8. Optical density (600 nm) of trans cinnamaldehyde MIC against test bacteria organisms. Data represents the means of triplicate analysis.

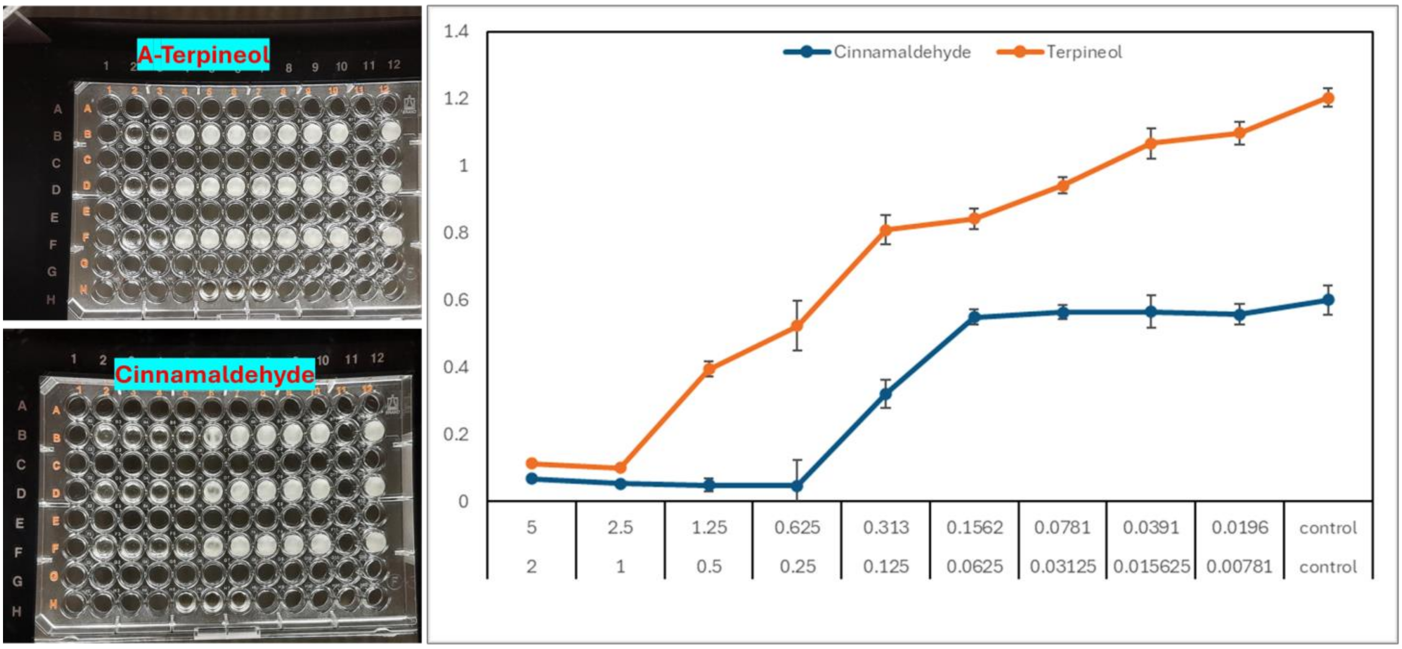

Figure S9. Optical density (600 nm) of trans cinnamaldehyde and A-Terpineol MIC against *C. albicans*. Data represents the means of triplicate analysis.

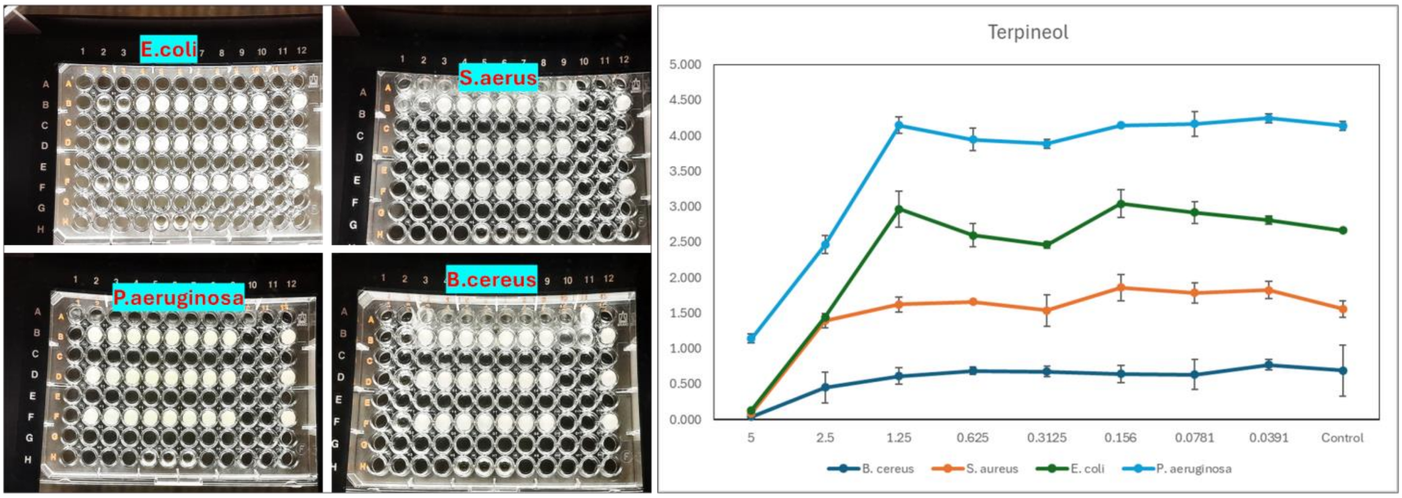

Figure S10. Optical density (600 nm) of α-terpineol against test bacteria organisms. Data represents the means of triplicate analysis.
